# Diastereoselective anodic hetero- and homo-coupling of menthol-, 8-methylmenthol- and 8-phenylmenthol-2-alkylmalonates

**DOI:** 10.3762/bjoc.13.5

**Published:** 2017-01-05

**Authors:** Matthias C Letzel, Hans J Schäfer, Roland Fröhlich

**Affiliations:** 1 Organisch-Chemisches Institut der Westfälischen Wilhelms- Universität, Corrensstraße 40, D-48149 Münster, Germany

**Keywords:** anodic decarboxylation, diastereoselectivity, Kolbe electrolysis, radical hetero-coupling, radical homo-coupling

## Abstract

Diastereoselective radical coupling was achieved with chiral auxiliaries. The radicals were generated by anodic decarboxylation of five malonic acid derivatives. These were prepared from benzyl malonates and four menthol auxiliaries. Coelectrolyses with 3,3-dimethylbutanoic acid in methanol at platinum electrodes in an undivided cell afforded hetero-coupling products in 22–69% yield with a diastereoselectivity ranging from 5 to 65% de. Electrolyses without a coacid led to diastereomeric homo-coupling products in 21–50% yield with ratios of diastereomers being 1.17:2.00:0.81 to 7.03:2.00. The stereochemistry of the new stereogenic centers was confirmed by X-ray structure analysis and ^13^C NMR data.

## Introduction

Intermolecular radical additions with high diastereoselectivity have been described for a number of cases [1–9]. There are much fewer reports on intermolecular diastereoselective radical coupling reactions [10–16]. There are some examples of high diastereoselectivity in the coupling of radicals generated from azo compounds [10–11], in intramolecular coupling of radicals, that are obtained by photochemical activation [12–14], and in intermolecular coupling of radicals generated by anodic decarboxylation of carboxylic acids [15–16].

**Figure 1:**
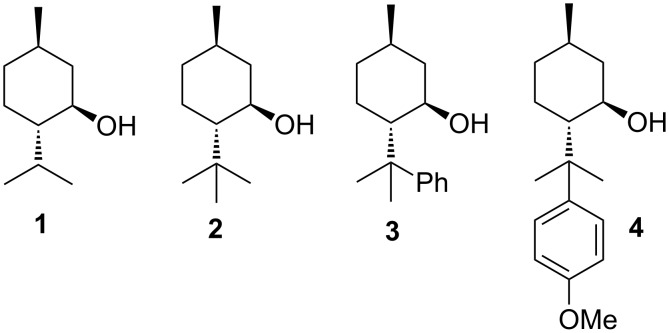
Menthol auxiliaries **1**–**4** used in the following anodic coupling reactions.

At the anode radicals can be generated by anodic decarboxylation of carboxylic acids in molar quantities, in a simple procedure, unaffected by cage effects and in large diversity. For that reason, we were interested in exploring the structural influence of chiral auxiliaries (Figure 1) and the carboxylic acid on the diastereoselectivity in the anodic coupling of carboxylic acids. We report here on the diastereoselectivity found in the anodic hetero- and homo-coupling of menthol- (**1**)-, 8-methylmenthol- (**2**)-, 8-phenylmenthol- (**3**)-, and 8-*p*-anisylmenthol- (**4**)-2-alkylmalonates (Figure 1).

## Results and Discussion

### Anodic hetero- and homo-coupling of carboxylates

**Scheme 1:**
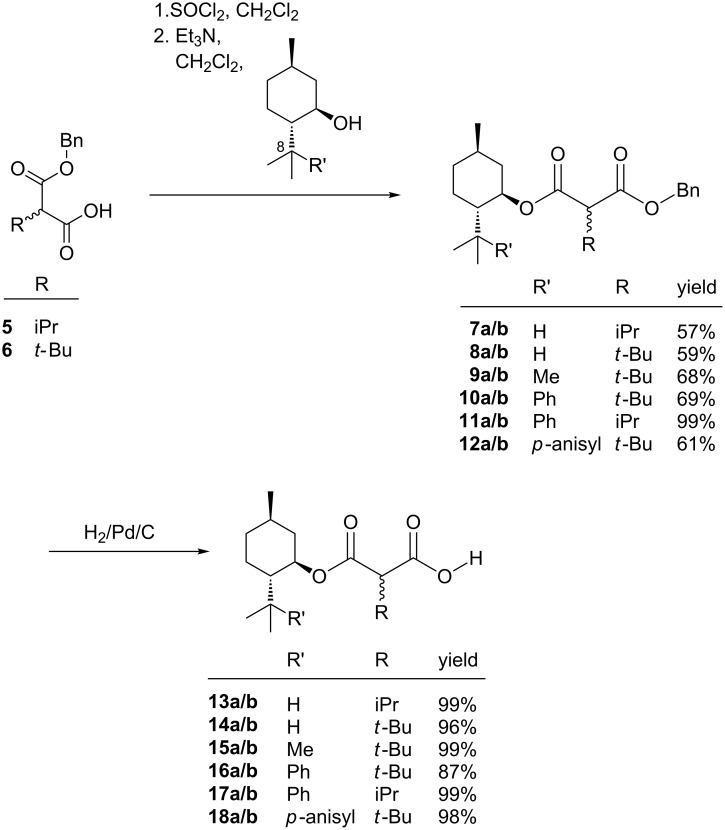
Synthesis of carboxylic acids **13a/b**–**18a/b**.

The carboxylic acids **13a**/**b**–**18a**/**b** for the Kolbe electrolyses were synthesized according to Scheme 1. The chiral auxiliary **1**–**4** is acylated with a benzylmalonyl chloride prepared from **5** and **6** and the benzyl group is subsequently removed by hydrogenation to afford the free carboxylic acids **13a/b**–**18a/b** for the electrolyses.

**Scheme 2:**
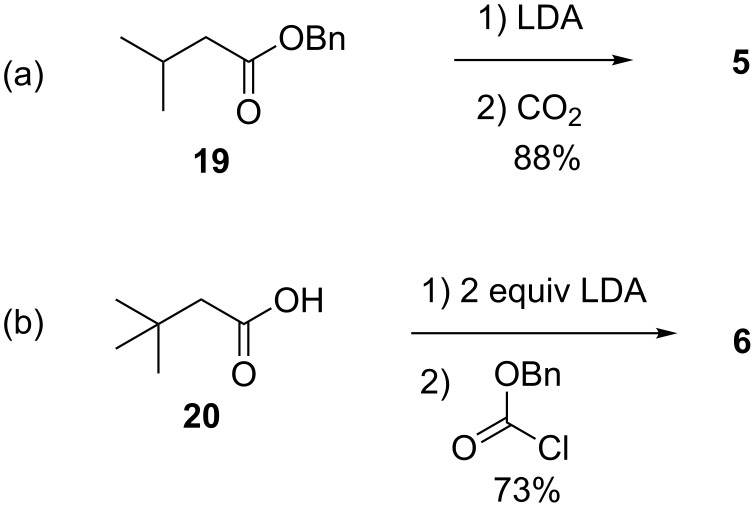
(a) Preparation of benzyl 2-isopropylmalonate (**5**) and (b) preparation of benzyl 2-*tert*-butylmalonate (**6**).

Benzyl 2-isopropylmalonate (**5**) was prepared in high yield, using a method of Strating et al. [17], by deprotonation of the benzyl ester **19** with lithium diisopropylamide and quenching the enolate with carbon dioxide (Scheme 2a). Benzyl 2-*tert*-butylmalonate (**6**) was prepared in good yield using a method of Krapcho et al. [18], by double deprotonation of 3,3-dimethylbutyric acid (**20**) with LDA and quenching the dianion with benzyl chloroformate (Scheme 2b).

The benzyl malonates **5** and **6** are converted to the corresponding benzyl malonyl chlorides with thionyl chloride. To these at −40 °C the auxiliary menthol (**1**), 8-*p*-anisylmenthol (**4**) [19], 8-methylmenthol (**2**) [20] or 8-phenylmenthol (**3**) [21] was added in the presence of triethylamine, whereby the menthyl esters **7**–**12** were obtained in good yield after flash chromatography as a mixture of two diastereomers (Scheme 1).

The benzyl menthyl malonates **7**–**12** were hydrogenated under atmospheric pressure to afford the free carboxylic acids **13a/b**–**18a/b** in high yield and purity (Scheme 1).

### Anodic hetero-coupling of carboxylates

Carboxylates of aliphatic carboxylic acids are oxidized to carbonyloxy radicals at a platinum electrode at potentials being generally higher than 2.0 V (vs NHE). The carbonyloxy radicals undergo fast decarboxylation to alkyl radicals at or near the electrode surface [22]. The generation of radicals in a thin reaction layer at the electrode surface leads to high radical concentrations that favor bimolecular radical coupling. Electrolysis of equal carboxylic acids leads to a symmetrical dimer by homo-coupling of the radicals. Coelectrolysis of two different carboxylic acids affords unsymmetrical coupling products by hetero-coupling. In the latter case three different coupling products are obtained: two symmetrical dimers and one unsymmetrical coupling product, whose ratio is in most cases determined by the statistical coupling of the intermediate radicals. An excess of one coacid leads to mainly two products: the unsymmetrical coupling product and the dimer of the coacid used in excess.

**Scheme 3:**
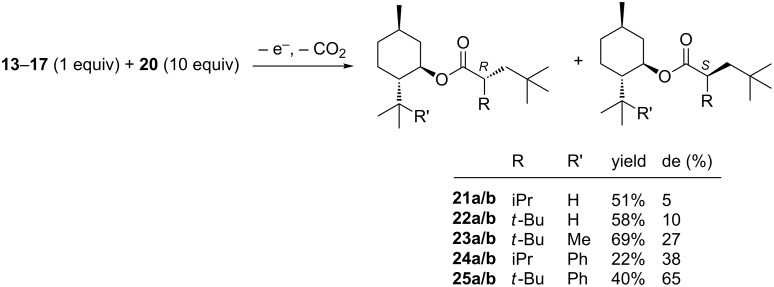
Coelectrolysis (hetero-coupling) of carboxylic acids **13**–**17** with 3,3-dimethylbutyric acid (**20**).

The coelectrolysis (hetero-coupling) of carboxylic acids **13**–**17** with ten equivalents of 3,3-dimethylbutyric acid (**20**) afforded the hetero-coupling products **21**–**25** in 22–69% yield of two diastereomers, whereby one of the two diastereomers was formed in 5–65% diastereomeric excess (Scheme 3).

Because of the low diastereomeric excess for compounds **21** and **22**, the configuration of the major diastereomer was not determined. The configurations for the major diastereomers of **23**–**25** were obtained from crystal structure analyses and the correlation of the ^13^C NMR data.

**Figure 2:**
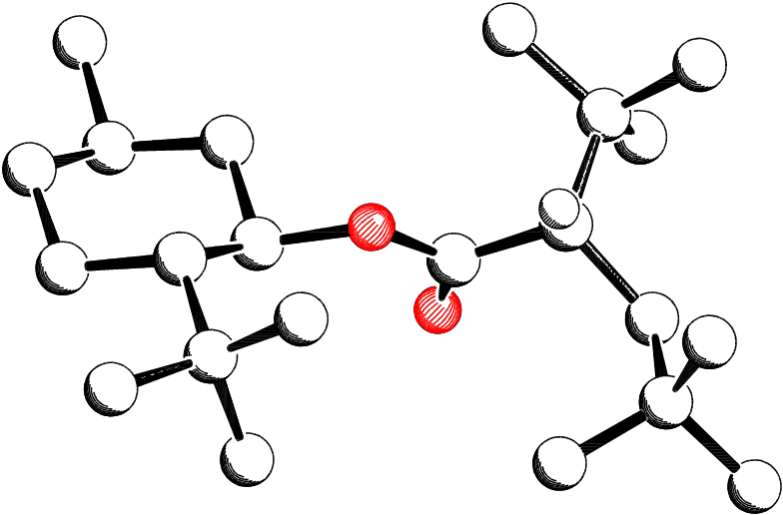
Crystal structure of the minor diastereomer **23b**.

According to an X-ray analysis the minor diastereomer **23b** has the configuration (*S*) at the new formed stereogenic center (Figure 2) [23].

The configuration of the new stereogenic centers in compounds **24** and **25** were assigned on the basis of a characteristic down field shift in the ^13^C NMR of the carbonyl and the methine carbon atom in the major diastereomer (Table 1).

**Table 1:** ^13^C NMR data of compounds **23**, **24** and **25**.

	^13^C NMR [ppm]			

No.	major diastereomer		minor diastereomer	
	*C*=O	*C*HCH_2_*t-*Bu	*C*=O	*C*HCH_2_*t-*Bu

**23**	176.5 (**23a**)	52.4 (**23a**)	175.1 (**23b**)	51.5 (**23b**)
**24**	176.4 (**24a**)	47.3 (**24a**)	175.1 (**24b**)	46.5 (**24b**)
**25**	176.7 (**25a**)	51.7 (**25a**)	175.1 (**25b**)	50.9 (**25b**)

The higher diastereomeric excess in compounds **24** and **25** obtained with 8-phenylmenthol (**3**) as auxiliary is attributed to an electronic interaction between the carbonyl and the phenyl group [24]. In order to enhance this interaction, the phenyl group was replaced for the more electron-rich anisyl group by applying 8-anisylmenthol (**4**) as chiral auxiliary. However, with acetic acid or 3,3-dimethylbutyric acid (**20**) as coacid no radical hetero-coupling product of carboxylic acid **18** was found, but polar compounds were obtained, whose structures were not determined.

**Figure 3:**
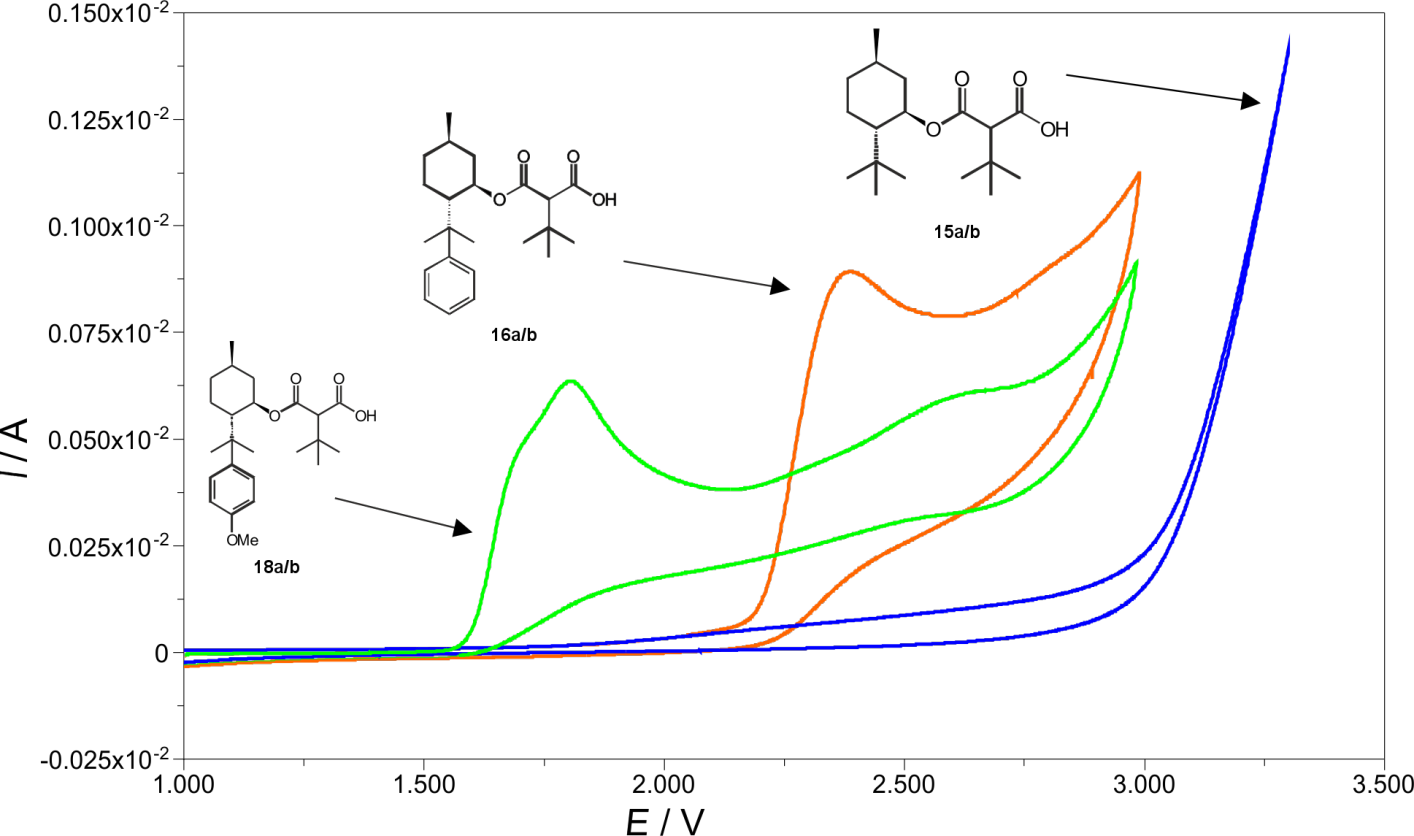
Cyclic voltammograms of the malonic derivatives **15a/b**, **16a/b** and **18a/b** (scan rate: 500 mA/s, solvent: acetonitrile, supporting electrolyte: tetrabutylammonium perchlorate (TBAClO_4_), reference electrode: Ag/AgCl).

To explain this failure, it is assumed that under the electrolysis conditions the electron-rich aromatic ring system is preferentially oxidized. Cyclic voltammetry of the malonates **15a/b**, **16a/b** and **18a/b** supports this assumption. The cyclic voltammogram of **18a/b** shows an oxidation peak at 1.75 V to 1.85 V (Figure 3).

In the cyclic voltammogram of malonic acid derivative **16a/b** a peak appears at a potential of 2.4 V (Figure 3). The malonic acid derivative **15a/b** does not show any oxidation peak below 3 V (Figure 3). In this case the increase in current at 3 V is caused by the oxidation of the solvent. These results clearly attribute the peak at 1.75 to 1.85 V in carboxylic acid **18a/b** to the oxidation of the anisyl group. This is also in accord with oxidation potentials from the literature [25], where the oxidation potential for anisole and toluene was determined to be 1.15 V and 1.35 V (vs Ag/Ag^+^), respectively.

In the Kolbe electrolysis a critical potential of 1.9 to 2.2 V (vs Ag/AgCl) has to be exceeded. At this potential the coverage of the electrode with carboxylate ions increases sharply, the oxygen evolution is inhibited, solvent oxidation is retarded and the Kolbe electrolysis is promoted [26].

If in the carboxylate an additional electrophore with a lower oxidation potential than the critical potential is present it is oxidized instead of the carboxylate group. This is the case for **18a/b**. Even in the case of **16a/b** and **17a/b** a significant portion of polar products is found, indicating a partial oxidation of the phenyl group.

### Anodic homo-coupling of carboxylates

**Scheme 4:**
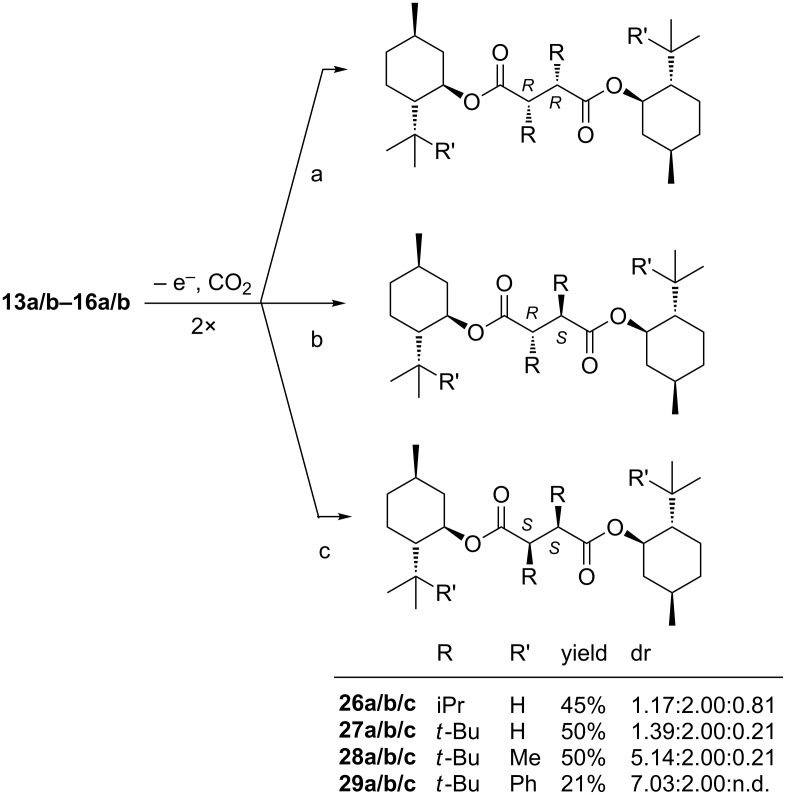
Homo-coupling of carboxylic acids **13a/b**–**16a/b** to diesters **26a/b/c**–**29a**/**b**/**c** (n.d.: not determined).

The carboxylic acids **13a/b**–**16a/b** have also been subjected to homo-coupling and the diastereomers **26a/b/c**–**29a/b/c** were afforded in 50–21% yield (Scheme 4).

In the homo-coupling reaction two identical radicals combine to form two stereogenic centers. Three diastereomers can be formed by *re,re*-, *re,si*- and *si,si*-coupling of the prostereogenic radical centers. Without facial selectivity they will be formed in a statistical ratio of 1:2:1. Facial selectivity is indicated by deviation from this statistical ratio. The selectivity for *re,re*-coupling vs *re,si*-coupling is given by the ratio 2 (**a**:**b**), the selectivity for *re,si*- vs *si,si*-coupling by **b**:2**c** (Table 2).

**Table 2:** Facial selectivities for **26**–**29**.

compound	*re,re:re,si*	*re,si:si,si*

**26**	1.17	1.23
**27**	1.39	1.49
**28**	5.14	4.76
**29**	7.03	not determinable

**Figure 4:**
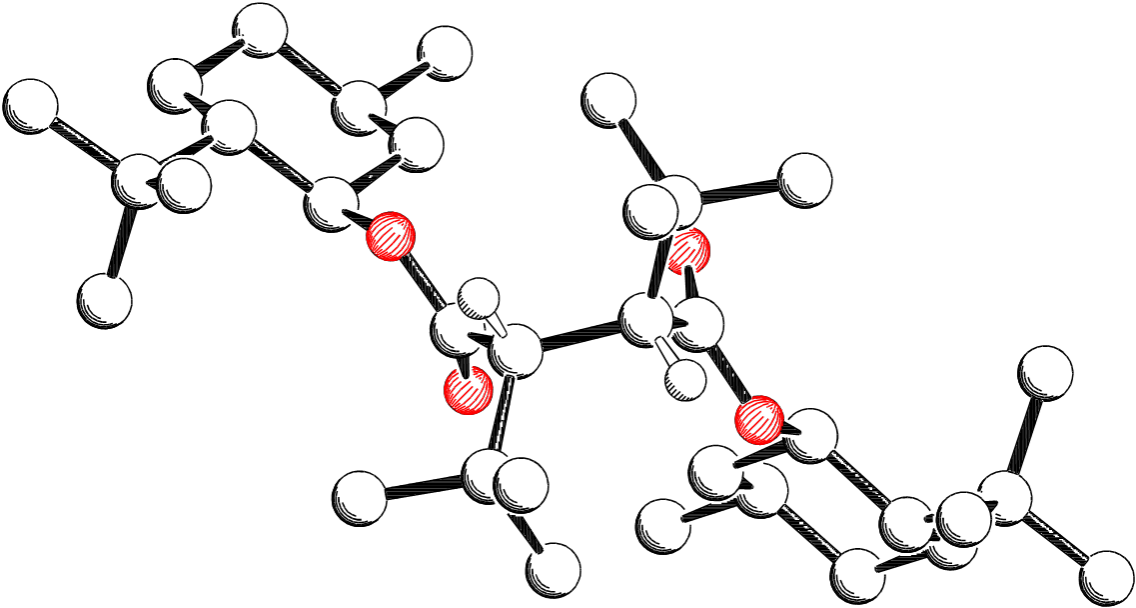
Crystal structure of major diastereomer **28a**.

**Figure 5:**
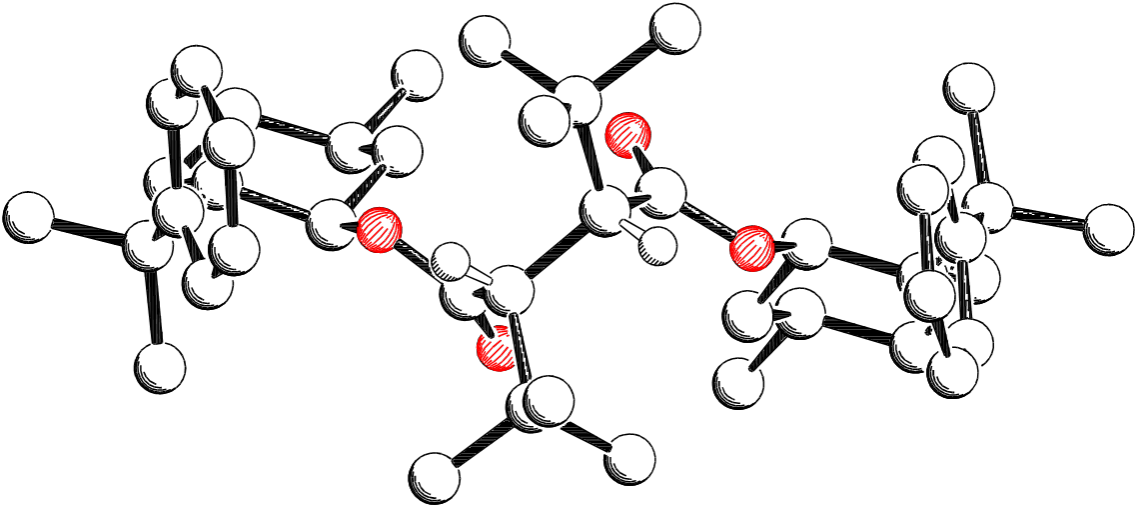
Crystal structure of major diastereomer **29a**.

The absolute configuration of the major diastereomers **28a** and **29a** was determined by X-ray analysis (Figure 4 [23] and Figure 5 [23], hydrogen atoms are left out, except those formed at the new stereogenic centers). The new stereogenic centers in both cases have (2’*R*,3’*R*)-configuration. For the diastereomers **26b**, **27b**, **28b** and **29b** a set of ^1^H and ^13^C NMR signals can be observed for the protons and the carbon atoms of each half of the molecule. Based on this observation a (2’*R*,3’*S*)-configuration was assigned to **26b**, **27b**, **28b** and **29b**. For the diastereomers **26a/c**, **27a/c**, **28a** and **29a** only a single set of signals is obtained, because both halves of the molecule are chemically equivalent due to its *C*_2_-symmetry.

In Figure 4 and Figure 5 the *C*_2_-symmetry can be easily seen. From Figure 2, Figure 4 and Figure 5 the conformation of the radicals that undergo the coupling reaction can be deduced. Assuming a homolytic cleavage of the bond between C2’–C3’ in compound **23b** (Figure 2) and converting the sp^3^ hybridized carbon atoms C2’ and C3’ into sp^2^ hybrids shows that the minor stereoisomer is formed by the shielding of the *re*-face through the *tert*-butyl group. Hence the major diastereomer **23a** is formed by an attack via the less shielded *si*-face.

The same consideration for compound **28a** (Figure 4) and compound **29a** (Figure 5) with a cleavage of the C2’–C3’ bond in the succinic acid derivatives shows that the major diastereomer is formed via the *si*-face, which is less shielded by the *tert*-butyl or cumyl group.

## Discussion

8-Phenylmenthol has been successfully used as a chiral auxiliary [27] to control the stereochemistry in Diels–Alder reactions [28], [2 + 2]- and [3 + 2]-cycloadditions and asymmetric ene reactions [29]; furthermore, it has been applied in 1,4-cuprate additions [30], Grignard additions [31], in supramolecular chemistry [32] and in alkylation of malonic acid derivatives [33]. In the literature, there are only a few examples where menthol auxiliaries have been used to control the stereochemistry in radical reactions [34].

In first experiments using (−)-menthol (**1**) as a chiral auxiliary we observed a low but nevertheless promising preference for one diastereomer, depending on the substituent R (Scheme 3). With the *tert*-butyl group in **22a/b** a higher selectivity was observed than with the isopropyl group in **21a/b**.

**Figure 6:**
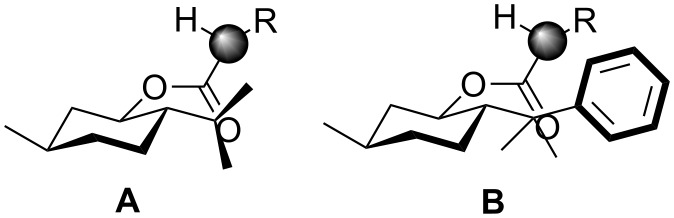
Discrimination of diastereomeric faces in the menthol substituted radical **A** and in the 8-phenylmenthol substituted radical **B**.

Still higher discrimination between both diastereomeric faces had been expected using a modified menthol with a phenyl group in 8-position, which is 8-phenylmenthol (Figure 6).

This hypothesis was proven to be accurate, obtaining an astonishingly high diastereoselectivity of 65% de for **25a/b** with R = *tert*-butyl and 8-phenylmenthol (**3**) (Scheme 3). In the same coupling reaction using 2,5-dimethylpyrrolidine as auxiliary the analogous coupling product was obtained with a selectivity of 65% de as well [15]. The selectivity decreases to 38% de for **24a/b** with R = isopropyl. When 8-methylmenthol (**2**) was used, **23a/b** was obtained with a moderate selectivity of 27% de, but a good yield of 69%.

The facial selectivity for a single radical center can be calculated from the diastereomeric ratio obtained in the homo-coupling reactions, assuming that there is no simple diastereoselectivity favoring either the (2*R*,3*R*)/(2*S*,3*S*)- or the (2*R*,3*S*)-diastereomers (Table 3). In coupling reactions without auxiliary control, no significant preference for either *d, l* or *meso* had been observed [35], proving this precondition to be legitimate.

**Table 3:** Comparison of the diastereoselectivity in hetero- and homo-coupling reactions.

Electrolysis	Homo-coupling	Hetero-coupling^a^
of compound	facial diastereoselectivity *re*:*si*	diastereomeric ratio *R*:*S*

**13a/b**	54.5:45.5^b^	52.5:47.5^b^
**14a/b**	59.0:41.0^b^	55.0:45.0^b^
**15a/b**	83.5:16.8	63.5:36.5
**16a/b**	87.5:12.5	82.5:17.5

^a^3,3-Dimethylbutanoic acid was used as coacid in all cases. ^b^The absolute configuration of the major diastereomer has not been determined. Therefore, the facial stereoselectivity is assumed to have the same preference as in the cases where the configuration is known.

Comparing the stereoselectivity in the homo- and hetero-coupling reaction (Table 3), a slightly better selectivity is observed for the homo-coupling reaction. This is probably due to the bulkier coradical in the homo-coupling, compared with the 2,2-dimethylpropyl radical in the hetero-coupling reaction.

With auxiliaries like 2,5-dimethylpyrrolidine, 2,5-dimethoxymethylpyrrolidine or camphorsultam side products resulting from decarboxylation, disproportionation and further oxidation of the intermediate radical have been observed [35–37]. In contrast, hardly any side products of that kind were obtained using menthol related auxiliaries in anodic radical coupling reactions.

Products analogous to these of the radical homo-coupling above can be obtained by auxiliary controlled oxidative enolate coupling in high yield with good stereocontrol [38–40]. With the auxiliary controlled alkylation of enolates products being analogous to radical hetero-coupling can be synthesized [41–42]. However, both methods require costlier reaction conditions as inert gas, dry solvents and LDA as reagent, and are probably harder to perform in a large scale as the Kolbe electrolysis.

There is strong evidence that in the Kolbe electrolysis free radicals are involved in the coupling reaction. Coupling of absorbed radicals is ruled out by Eberson [43–45]. It was shown that the product ratio of cyanoalkyl radicals, which can yield C–C or C–N coupling products, does not differ in the Kolbe electrolysis from the ratio obtained by the coupling of photochemically produced cyanoalkyl radicals. Optically active carboxylates, which have the asymmetric center at C2 racemize in mixed Kolbe electrolyses [46–47]. This finding points to a free radical or its reversible adsorption at the electrode. However, the latter pathway was ruled out by experiments of Utley et al. [48–49], when comparing the coupling of unsaturated and saturated carboxylic acids and the effect of adsorption.

**Scheme 5:**
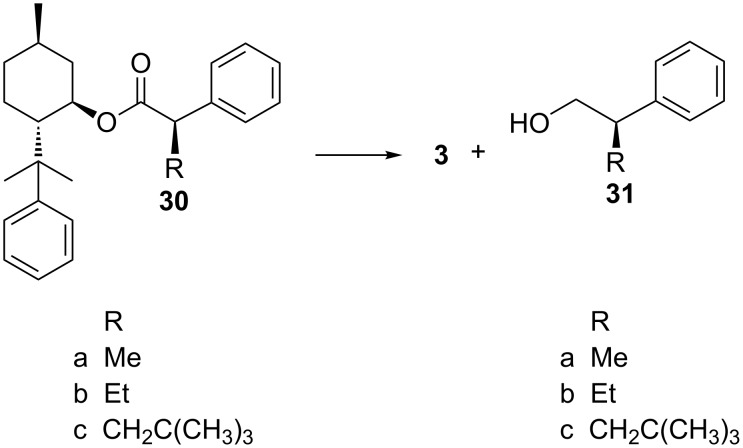
Reductive cleavage of **30a–c** to 8-phenylmenthol (**3**) and **31a–c**.

Ester **30a** has been cleaved with lithium aluminium hydride in THF to **3** and **31a** in 90% (Scheme 5) and with **30b** (Scheme 5) and DIBAL in toluene 92% of **31b** were obtained [50]. **30c** could be cleaved with lithium aluminium hydride in THF to 83% **3** and 90% **31c** [51]. However, to our great disappointment the successful cleavage with **30c** could not be transferred to the structurally similar compounds **23a/b** and **25a/b**. Using the same reaction conditions as with **30a** both with lithium aluminium hydride in THF and DIBAL in toluene with **23a/b** and **25a/b** the starting material was completely reisolated. Apparently the carbonyl group is so much shielded by the *tert-*butyl group and the auxiliary that the reducing agent is not able to reach the carbonyl group.

However, the phenyl group at C2 of the ester **30c** causes lower yields (11%) of anodic coupling product, because it facilitates the oxidation of the intermediate radical and also lowers the diastereoselectivity significantly (de 39%) compared to the bulkier *tert-*butyl group with 65% de in **25a/b** (Scheme 3) [51]. Therefore, the *tert-*butyl group appears to be important for a higher product yield and the diastereoselectivity of the coupling. On the other side this bulky group prohibits the cleavage with the so far successful reagents LiAlH_4_ and DIBAL. Therefore, in the future bulky chiral auxiliaries must be developed that can be partially disassembled before their cleavage and slim cleavage reagents have to be found and explored.

## Conclusion

We developed an efficient procedure for the synthesis of menthyl 2-*tert-*butyl and 2-isopropyl-4,4-dimethylpentanoates (**21a/b**–**25a/b**) with a chiral centre at C2 of the acid component. For that purpose, malonic half-esters with substituted menthols as chiral auxiliaries were prepared. The half-esters were electrochemically decarboxylated to alkyl radicals. In the presence of an excess of 3,3-dimethylbutylbutanoic acid that decarboxylated to a 2,2-dimethylpropyl radical preferentially the hetero-coupling products **21a/b**–**25a/b** were obtained in up to 69% yield and diastereomeric ratios of up to 82.5:17.5 induced by the menthyl auxiliaries. However, the removal of the auxiliary in the esters is not yet resolved. Without a coacid succinic acid derivatives **26a/b/c**–**29a/b/c** were obtained by dimerization of the half-ester radicals with yields of up to 50% and a facial diastereoselectivity of up to 87.5:12.5.

Products that are similar to these of the anodic coupling can be obtained by auxiliary controlled oxidative enolate coupling. However, this method often requires more expensive reagents, functional groups are frequently more sensible to the used reagents and an up-scale is more difficult to perform.

On the other side anodic coupling of sodium carboxylates is easy and simply to do. Methanol can be mostly used as solvent, the alkali salt of the carboxylic acid serves at the same time as substrate and supporting electrolyte. The required equipment is simple, inexpensive and readily available. An undivided beaker-type cell is sufficient; electrolysis can be performed current-controlled with a cheap power supply and an up-scale of the reaction is mostly easy.

## Experimental

### General remarks

Optical rotations: Perkin-Elmer polarimeter 241, specific rotations in grad·cm^3^·dm^−1^·g^−1^, *c* in g/100 mL. FTIR: Bruker IFS 28. ^1^H, ^13^C NMR: Bruker WM 300. X-ray structures: Data sets were collected with an Enraf Nonius CAD4 diffractometer. Programs used: data reduction MolEN, structure solution SHELXS-86, structure refinement SHELXL-93, graphics SCHAKAL-92. MS: Finnigan MAT, Varian Saturn II (ion trap) with capillary column HP 5 (25 m, 0.2 mm i.d., 0.33 μm film); DCI with ammonia as reactant gas. GLC: Hewlett-Packard HP 5890 Series II, Shimadzu GC-14A, Carlo Erba 4200; quartz capillary columns: Macherey and Nagel FS-HP1 (25 m, 0.32 mm i.d., 0.25 μm film), AT 35 (2.5 m, 0.32 mm i.d., 0.25 μm film). Preparative scale electrolyses: Undivided beaker-type glass cell, double walled for constant temperature control, capacity: 3 mL (micro beaker-type cell). Platinum foils serving as anode and cathode materials (platinum foils: 1 × 1 cm). Convection was achieved by magnetic stirring. Current source: Heinzinger galvanostat – potentiostat HN 600–600 and TNs 300–1500.

### General procedure for the Kolbe electrolysis

**Hetero-coupling reaction:** 0.4 mmol of the auxiliary modified carboxylic acid and 4 mmol of the coacid were dissolved in 2.2 mL of a 0.1 N methanolic solution of sodium methanolate (5% neutralisation) and transferred into the micro-beaker-type cell. Dry methanol was added to a total volume of 3 mL, which was electrolyzed maintaining a current density of 400 mA/cm^2^ and the temperature of the solution in the range of 5–10 °C with a coolant of −25 °C. When the pH of the solution reached 8–9 the electrolysis was stopped, the mixture was poured into 30 mL of water and extracted with ether (5 × 20 mL). The combined organic layers were washed with saturated sodium hydrogen carbonate (2 × 20 mL) and dried over MgSO_4_. After evaporation of the solvent in the vacuum the product was purified by flash chromatography.

**Homo-coupling reaction:** 1.6 mmol of the auxiliary modified carboxylic acid was dissolved in 1.6 mL of a 0.1 N methanol solution of sodium methoxide (5% neutralization) and transferred into the micro-beaker-type cell. Dry methanol was added to a total volume of 3 mL, which was electrolyzed maintaining a current density of 400 mA/cm^2^ and the temperature of the solution in the range of 5–10 °C with a coolant of −25 °C. The further work-up follows the procedure given for the hetero-coupling reaction.

**General procedure for the preparation of the menthol esters:** To a solution of 4.3 mmol of monobenzyl malonate **5** or **6** in 20 mL dry methylene chloride 0.8 mL thionyl chloride (1.3 g, 10.8 mmol) were added and the mixture was refluxed for 3 h. After evaporation of the solvent and the unreacted thionyl chloride in vacuum, the remaining yellow oil was dissolved in 20 mL methylene chloride without further purification and cooled to −40 °C. Under an argon atmosphere 2.81 mmol auxiliary, dissolved in 10 mL dry methylene chloride and 10 mL dry triethylamine, were added slowly to the carboxylic acid chloride at −40 °C. After the complete addition, the mixture was allowed to warm up to rt and stirred for 20 h. Then 20 mL of water were added and the mixture was extracted with methylene chloride (4 × 20 mL). The combined organic layers were washed successively with 2 N HCl (3 × 20 mL), saturated sodium hydrogen carbonate (2 × 20 mL) and brine (20 mL) and dried (MgSO_4_). After evaporation of the solvent the product was purified by flash chromatography.

**General procedure for the hydrogenation of the benzyl esters:** To a solution of 6.4 mmol benzyl ester in 100 mL of methanol 800 mg of the palladium catalyst (10% Pd on carbon) were added. Hydrogenation was performed at atmospheric pressure until no further hydrogen was consumed. After filtration through celite the solvent was evaporated in vacuum. The residue was dissolved in a small amount of diethyl ether, filtrated through celite and dried with MgSO_4_. After evaporation of the solvent the free carboxylic acid was obtained with high purity as a sticky oil.

The preparation of compounds **5**–**29**, their spectroscopic data and their elemental analyses are reported in Supporting Information File 1.

## Supporting Information

File 1 Experimental part.
