# Supplementary material for: Diastereoselective anodic hetero- and homo-coupling of menthol-, 8-methylmenthol- and 8-phenylmenthol-2-alkylmalonates
Source: Beilstein J Org Chem. 2017 Jan 5;13:33–42. doi: 10.3762/bjoc.13.5 (PMC5238534; doi:10.3762/bjoc.13.5)
Supplement: File 1 — Experimental part. [file Beilstein_J_Org_Chem-13-33-s001.pdf]

Supporting Information

for

Diastereoselective anodic hetero- and homo-coupling of menthol-, 8-methylmenthol- and 8-phenylmenthol-2-alkylmalonates

Matthias C. Letzel, Hans J. Schäfer* and Roland Fröhlich§

Organisch-Chemisches Institut der Westfälischen Wilhelms-Universität, Corrensstraße 40, D-48149 Münster, Germany

*Corresponding author

Email: Hans J. Schäfer - schafeh@uni-muenster.de

§X-ray structure analysis

Experimental part

Table of Contents

1. *2-((Benzyloxy)carbonyl)-3-methylbutanoic acid* (**5**): S3
2. *2-((Benzyloxy)carbonyl)-3,3-dimethylbutanoic acid* (**6**): S4
3. *1-Benzyl 3-[(1R,2S,5R)-2-isopropyl-5-methylcyclohexyl] (2´R/S)-2´-isopropylmalonate* (**7a/b**): S5
4. *1-Benzyl 3-[(1R,2S,5R)-2-isopropyl-5-methylcyclohexyl] (2´R/S)-2´-(tert-butyl)malonate* (**8a/b**) S6
5. *1-Benzyl 3-[(1R,2S,5R)-2-(tert-butyl)-5-methylcyclohexyl] (2´R/S)-2´-(tert-butyl)malonate* (**9a/b**) S6
6. *1-Benzyl 3-[(1R,2S,5R)-2-(2-phenylpropan-2-yl)-5-methylcyclohexyl] (2´R/S)-2´-(tert-butyl)malonate* (**10a/b**) S7
7. *1-Benzyl 3-[(1R,2S,5R)-2-(2-phenylproan2-yl)-5-methylcyclohexyl] (2´R/S)-2´-isopropylmalonate* (**11a/b**) S8

8. *1-Benzyl 3-{(1R,2S,5R)-2-[2-(4-methoxyphenyl)propan-2-yl)]-5-methylcyclohexyl} (2´R/S)-2´-(tert-butyl)malonate* (**12a/b**) S9

9. *2´-[(1R,2S,5R)-2-Isopropyl-5-methylcyclohexyloxycarbonyl] (2´R/S)-3´-methylbutanoic acid* (**13a/b**) S10

10. *2´-[(1R,2S,5R)-2-Isopropyl-5-methylcyclohexyloxycarbonyl] (2´R/S)-3´,3´-dimethylbutanoic acid* (**14a/b**) S10

11. *2´-[(1R,2S,5R)-2-(tert-Butyl)-5-methylcyclohexyloxycarbonyl] (2´R/S)-3´,3´dimethylbutanoic acid* (**15a/b**) S11

12. *3´,3´-Dimethyl-2´-[(1R,2S,5R)-5-methyl-2-(2-phenylpropan-2-yl)cyclohexyloxycarbonyl] (2´R/S)-butanoic acid* (**16a/b**) S12

13. *3´-Methyl-2´-[(1R,2S,5R)-2-(1-Methyl-1-phenylethyl)-5-methylcyclohexyloxycarbonyl)] (2´R/S)-butanoic acid* (**17a/b**) S12

14. *2´-(1R,2S,5R)-2-[2-(4-Methoxyphenyl)propan-2-yl)-5-methylcyclohexyloxycarbonyl] (2´R/S)-3´3´-dimethylbutanoic acid* (**18a/b**) S13

15. *(1R,2S,5R)-2-Isopropyl-5-methylcyclohexyl (2´R/S)-2´-isopropyl-4,4-dimethyl-pentanoate* (**21a/b**) S14

16. *(1R,2S,5R)-2-Isopropyl-5-methylcyclohexyl (2´R/S)-2´-(tert-butyl)-4´,4´-dimethylpentanoate* (**22a/b**) S14

17. *(1R,2S,5R)-2-(tert-Butyl)-5-methylcyclohexyl (2´R)-2´-(tert-butyl)-4´,4´-dimethyl-pentanoate* (**23a**) and *(1R,2S,5R)-2-(tert-butyl)-5-methylcyclohexyl (2´S)-2´-(tert-butyl)-4´,4´-dimethylpentanoate* (**23b**) S15

18. *(1R,2S,5R)-5-Methyl-2-(2-phenylpropan-2-yl)cyclohexyl (2´R)-2´-isopropyl-4´,4´-dimethylpentanoate* (**24a**) and *(1R,2S,5R)-5-methyl-2-(2-phenylpropan-2-yl)cyclohexyl (2´S)-2´-isopropyl-4´,4´-dimethylpentanoate* (**24b**) S16

19. *(1R,2S,5R)-5-Methyl-2-(2-phenylpropan-2-yl)cyclohexyl (2´R)-2´-(tert-butyl)-4´,4´-dimethylpentanoate* (**25a**) and *(1R,2S,5R)-5-methyl-2-(2-phenylpropan-2-yl)cyclohexyl (2´S)-2´-(tert-butyl)-4´,4´-dimethylpentanoate* (**25b**) S17

20. *Bis[(1R,2S,5R)-2-isopropyl-5-methylcyclohexyl] (2´R*,3´R*)-2´,3´-diisopropyl-succinate* (**26a**), *bis[(1R,2S,5R)-2-isopropyl-5-methylcyclohexyl] (2´R,3´S)-2´,3´-diisopropylsuccinate* (**26b**) and *bis[(1R,2S,5R)-2-isopropyl-5-methylcyclohexyl] (2´S*,3´S*)-2´,3´-diisopropylsuccinate* (**26c**) S18

21. *Bis[(1R,2S,5R)-2-isopropyl-5-methylcyclohexyl] (2´R*,3´R*)-2´,3´- di(tert-butyl)-succinate* (**27a**), *bis[(1R,2S,5R)-2-isopropyl-5-methylcyclohexyl] (2´R,3´S)-2´,3´-di(tert-butyl)succinate* (**27b**) and *bis[(1R,2S,5R)-2-isopropyl-5-methylcyclohexyl] (2´S*,3´S*)-2´,3´- di(tert-butyl)succinate* (**27c**) S19

22. *Bis[(1R,2S,5R)-2-(tert-butyl)-5-methylcyclohexyl] (2´R,3´R)-2´,3´-di(tert-butyl)succinate* (**28a**) and *bis[(1R,2S,5R)-2-(tert-butyl)-5-methylcyclohexyl] (2´R,3´S)-2´,3´-di(tert-butyl)succinate* (**28b**) S21

23. *Bis[(1R,2S,5R)-5-Methyl-2-(2-phenylpropan-2-yl)cyclohexyl] (2´R,3´R)-2´,3´-di(tert-butyl)succinate* (**29a**) and *bis[(1R,2S,5R)-5-Methyl-2-(2-phenylpropan-2-yl)cyclohexyl] (2´R,3´S)-2´,3´-di(tert-butyl)succinate* (**29b**) S22

24. *(1R,2S,5R)-5-Methyl-2-(2-phenylpropan-2-yl) cyclohexyl (2R)-4,4-dimethyl-2-phenylpentanoate (R-**30c**)* S23

25. *X-ray crystal structure analysis of* **23b**, **28a** and **29a**: S25

1. *2-((Benzyloxy)carbonyl)-3-methylbutanoic acid* (**5**):

160 mL (250 mmol, 1.6 M in *n*-hexane) *n*-butyllithium were added to a solution of 35.14 mL (25.3 g, 250 mmol) of dry diisopropylamine in 250 mL dry THF at −70 °C. Finally, 41.2 g

(210 mmol) benzyl 3-methylbutanoate, dissolved in 170 mL THF, were added. After complete addition a stream of dry carbon dioxide was bubbled through the solution for about 20 min. At –70 °C the reaction mixture was carefully quenched with 40 mL of water and warmed up to rt. After acidification with conc. HCl the mixture was extracted with diethyl ether (3 × 150 mL). The combined organic layers were extracted with saturated sodium hydrogen carbonate (5 × 70 mL). The combined aqueous layers were washed with 70 mL of ether and then acidified with conc. HCl to pH 1. The acidic solution was extracted with ether (4 × 100 mL), and the combined extracts were dried over $MgSO_4$. After the evaporation of the solvent 44.68 g (189.1 mmol, 88%) monoester **5** was obtained as an oily liquid.

FT-IR (film): ν = 1732, 1714 cm^{-1}. — ^{1}H-NMR ($CDCl_3$): δ = 1.00, 1.04 (2d, *J* = 6.7, each 3H, $CH(CH_3)_2$), 2.41 (dsept, *J* = 6.9 Hz, *J* = 6.7 Hz, 1H, C*H*($CH_3)_2$), 3.24 (d, *J* = 6.9 Hz, 1H, C*H*$CH(CH_3)_2$), 5.17 (d, *J* = 12.2 Hz, 1H, PhCH_aH_b), 5.22 (d, *J* = 12.2 Hz, 1H, PhCH_aH_b), 7.30–7.40 (m, 5H, phenyl). — ^{13}C-NMR ($CDCl_3$): δ = 20.22, 20.32 (2q, CH(*C*$H_3)_2$), 29.25 (d, *C*H($CH_3)_2$), 45.94 (d, *C*H$CH(CH_3)_2$), 67.23 (t, Ph*C*H_2O), 128.26, 128.43, 128.60 (3d, =*C*-H_{phenyl}), 135.27 (s, =*C*–C_{phenyl}), 168.93 (s, C=O_{ester}), 173.55 (s, C=O_{acid}). — MS (GC/MS, **5** as methylester), *m/z* (%): 250 (19), 222 (14), 219 (4), 143 (14), 127 (3), 116 (86), 107 (18), 101 (52), 91 (100), 69 (17), 65 (10), 59 (18). — $C_{13}H_{16}O_4$ (236.3): calcd. C 66.09, H 6.83; found C 66.10, H 6.86.

2. *2-((Benzyloxy)carbonyl)-3,3-dimethylbutanoic acid* (**6**):

125 mL *n*-butyllithium (200 mmol, 1.6 M in *n*-hexane) were added to a solution of 20.2 g (0.2 mol, 28.1 mL) diisopropylamine in 500 mL dry THF at –40 °C. The solution was stirred for 15 min and then 11.6 g (100 mmol, 13 mL) *tert*-butylacetic acid were added. The mixture was heated at 50 °C for 1 h. After cooling to −70 °C 17.1 g (100 mmol, 14.3 mL) benzyl chloroformate were added. The solution was stirred for an additional 20 min., then poured on

ice and the mixture was then acidified with 30 mL (0.3 mol) conc. HCl. The aqueous layer was extracted with ether (6 ×150 mL). After evaporation of the solvent a yellow oil was obtained. The oil was treated with saturated sodium hydrogen carbonate and washed with ether (3 × 40 mL) for purification. The aqueous layer was acidified with conc. HCl to pH 1 with cooling. Extraction with ether (5 × 70 mL), drying with $MgSO_4$ and evaporation of the solvent yielded the yellow, oily product **6** (18.27 g, 73.0 mmol, 73%).

FT-IR (film): ν = 1737, 1713 cm^{-1}. — ^{1}H-NMR ($CDCl_3$): δ = 1.12 (s, 9H, $C(CH_3)_3$), 3.32 (s, 1H, C*H*tBu), 5.15, 5.20 (2d, *J* = 12.2 Hz, 2H, PhCH_aH_b), 7.25–7.40 (m, 5H, C_6H_5). — ^{13}C-NMR ($CDCl_3$): δ = 28.0 (q, C($C$$H_3$)$_3$), 34.2 (s, *C*$(CH_3)_3$), 61.1 (d, *C*H*t*Bu), 70.1 (t, O*C*H_2Ph), 128.3, 128.4, 128.6 (d, =*C*-H_{phenyl}), 135.2 (s, =*C*–C_{phenyl}), 168.8 (s, C=O_{ester}), 173.3 (s, C=O_{acid}). — MS (GC/MS, ion trap, **6** as methylester), *m/z* (%): 264 (3), 157 (1), 130 (35), 115 (45), 101 (28), 91 (100), 65 (16), 57 (12). — HR MS (EI): $C_{14}H_{18}O_4$: calcd. 250.1205; found 250.1208.

3. *1-Benzyl 3-[(1R,2S,5R)-2-isopropyl-5-methylcyclohexyl] (2´R/S)-2´-isopropylmalonate* (**7a/b**): 1.41 g (9.0 mmol) (–)-menthol and 4.25 g (18 mmol) monobenzyl ester **5** were converted according to the general procedure for the preparation of the menthol esters to afford 1.91 g (5.10 mmol, 57%) of carboxylic acid **7a/b** as a diastereomeric mixture after flash chromatography (petroleum ether/diethyl ether, 10:1).

FT-IR (film): ν = 1753, 1731 cm^{-1}. — ^{1}H-NMR ($CDCl_3$): δ = 0.68, 0.71 (2d, *J* = 6.9 Hz, in total 3H, 10-H), 0.80–2.00 (m, 9H, 2-H to 7-H), 0.99, 1.00 (2d, *J* = 6.7 Hz, in total 6H, CH(CH_3)$_2$), 0.83, 0.87, 0.88 (3d, *J* = 7.3 Hz or *J* = 6.4 Hz, in total 6H, 8-H, 9-H), 2.42 (dsept, 1H, C*H*$(CH_3)_2$), 3.151, 3.154 (2d, each *J* = 8.8 Hz, 1H, 2´-H), 4.69 (ddd, *J* = 4.5 Hz, *J* = 10.9 Hz, *J* = 10.9 Hz, 1H, 1-H), 5.10, 5.20 (2d, *J* = 12.4 Hz, 2H, CH_aH_bPh, **7a**), 5.12, 5.18 (2d, *J* = 12.2 Hz, 2H, CH_aH_bPh, **7b**), 7.25–7.40 (m, 5H, C_6H_5). — ^{13}C-NMR ($CDCl_3$): δ = 15.91 (q, C-

10), 20.32, 20.49 (2q, CH(*C*H_3)$_2$), 20.66, 21.94 (2q, C-8, C-9), 23.18 (t, C-3), 25.91, 26.02 (2d, C-5), 28.54, 28.68 (2d, *C*H(CH_3)$_2$), 31.31 (d, C-7), 34.17 (t, C-4), 40.37, 40.61 (2t, C-6), 46.71, 46.81 (2d, C-2), 59.34, 59.38 (2d, C-2´), 66.76 (t, O*C*H_2Ph), 75.32 (d, C-1), 128.19, 128.25, 128.49 (3d, =*C*-H_{phenyl}), 135.53 (s, =*C*–C_{phenyl}), 168.12, 168.25, 168.69 (3s, C=O). — MS (GC/MS), *m/z* (%): 374 (0.5), 359 (0.1), 236 (36), 208 (14), 138 (20), 107 (27), 95 (24), 91 (100), 83 (45). — $C_{23}H_{34}O_4$ (374.5): calcd. C 73.76, H 9.15; found C 73.86, H 9.45.

4. *1-Benzyl 3-[(1R,2S,5R)-2-isopropyl-5-methylcyclohexyl] (2´R/S)-2´-(tert-butyl)malonate* (**8a/b**): 1.25 g (8.0 mmol) (–)-menthol and 3.00 g (12 mmol) monobenzyl ester **6** were converted according to the general procedure for the preparation of the menthol esters to afford 2.16 g (5.56 mmol, 69%) **8a/b** as a diastereomeric mixture after flash chromatography (petroleum ether/diethyl ether, 20:1).

FT-IR (film): ν = 1753, 1730 cm^{-1}. — ^{1}H-NMR ($CDCl_3$): δ = 0.66, 0.71 (2d, *J* = 7.1 Hz, 3H, 10-H), 0.80–2.00 (m, 9H, 2-H to 7-H), 1.13 (s, 9H, C(CH_3)$_3$), 0.81, 0.83, 0.85, 0.88 (4d, *J* = 6.9 Hz, in total 6H, 8-H, 9-H), 3.26, 3.27 (2s, 1H, C*H*C(CH_3)$_3$), 4.68, 4.69 (2ddd, *J* = 4.3 Hz, *J* = 10.7 Hz, *J* = 10.7 Hz, 1H, 1-H), 5.08–5.18 (m, 2H, CH_aH_bPh), 7.25–7.38 (m, 5H, C_6H_5). — ^{13}C-NMR ($CDCl_3$): δ = 15.91 (q, C-10), 20.69, 21.94 (2q, C-8, C-9), 23.18 (t, C-3), 26.02 (d, C-5), 28.04 (q, C(*C*H_3)$_3$), 31.94 (d, C-7), 33.53, 33.66 (2s, *C*(CH_3)$_3$), 34.17 (t, C-4), 40.40, 40.71 (2t, C-6), 46.71, 46.84 (2d, C-2), 61.47, 61.63 (2d, *C*H*t*Bu), 66.52 (t, OCH_2Ph), 75.11 (d, C-1), 128.19, 128.29, 128.46 (3d, =*C*-H_{phenyl}), 135.69 (s, =*C*–C_{phenyl}), 167.75, 168.25 (2s, C=O). — MS (GC/MS), *m/z* (%): 388 (0.5), 373 (0.2), 250 (40), 193 (1), 138 (27), 107 (22), 95 (20), 91 (100), 83 (55), 57 (29). — $C_{24}H_{36}O_4$ (388.6): calcd. C 74.19, H 9.34; found C 73.88, H 9.53.

5. *1-Benzyl 3-[(1R,2S,5R)-2-(tert-butyl)-5-methylcyclohexyl] (2´R/S)-2´-(tert-butyl)malonate* (**9a/b**): 1.00 g (5.87 mmol) (–)-8-methylmenthol **2** and 1.78 g (7.11 mmol) monobenzyl ester **6** were converted according to the general procedure for the preparation of the menthol esters

to afford 1.62 g (4.02 mmol, 68%) **9a/b** as a diastereomeric mixture after flash chromatography (petroleum ether/diethyl ether, 10:1).

FT-IR (film): ν = 1751, 1730 cm^{-1}. — ^{1}H-NMR ($CDCl_3$): δ = 0.74 (d, *J* = 7.1 Hz, 3H, 11-H), 0.60–1.90 (m, 8H, 2-H to 6-H), 0.79 (s, 9H, 8-H to 10-H), 1.05, 1.06 (2s, 9H, $COC(CH_3)_3$), 3.13, 3.16 (2s, 1H, 2´-H), 4.67 (ddd, *J* = 4.3 Hz, *J* = 10.5 Hz, *J* = 10.5 Hz, 1H, 1-H), 5.01, 5.09, (2d, *J* = 12.2 Hz, 2H, CH_aH_bPh, **9b**, superposed by **9a**), 5.01, 5.11 (2d, *J* = 12.2 Hz, 2H, CH_aH_bPh, **9a**), 7.21–7.32 (m, 5H, C_6H_5). — ^{13}C-NMR ($CDCl_3$): δ = 21.77 (q, C-11), 26.45 (t, C-3), 28.10 (q, $C(CH_3)_3$), 28.98 (q, C-8 to C-10), 31.17 (d, C-5), 32.52 (s, C-7), 33.70 (s, $C(CH_3)_3$), 34.57 (t, C-4), 41.01, 41.65 (2t, C-6), 49.60, 49.81 (2d, C-2), 61.57, 61.94 (2d, C-2´), 66.65 (t, OCH_2Ph), 75.92, 76.29 (2d, C-1), 128.25, 128.46 (2d, =C-H_{phenyl}), 135.53 (s, =C–C_{phenyl}), 167.21, 168.32 (2s, C=O). — MS (GC/MS), *m/z* (%): 387 (0.2), 345 (0.3), 250 (10), 152 (5), 107 (6), 91 (100), 83 (9), 57 (37). — $C_{25}H_{38}O_4$ (402.6): calcd. C 74.59, H 9.51; found C 74.36, H 9.57.

6. *1-Benzyl 3-[(1R,2S,5R)-2-(2-phenylpropan-2-yl)-5-methylcyclohexyl] (2´R/S)-2´-(tert-butyl)malonate* (**10a/b**): 654 mg (2.81 mmol) (–)-8-phenylmenthol **3** and 1.08 g (4.31 mmol) monobenzyl ester **6** were converted according to the general procedure for the preparation of the menthol esters to afford 901 mg (1.94 mmol, 69%) **10a/b** as a diastereomeric mixture after flash chromatography (petroleum ether/diethyl ether, 10:1). An analytical sample of each diastereomer was separated by flash chromatography.

FT-IR (film): ν = 1748, 1729 cm^{-1}. — ^{1}H-NMR ($CDCl_3$): **10a**: δ = 0.60–2.00 (m, 8H, 2-H to 6-H), 0.72 (d, *J* = 6.4 Hz, 3H, 10-H), 1.09 (s, 9H, $C(CH_3)_3$), 1.19, 1.27 (2s, each 3H, 8-H, 9-H), 2.58 (s, 1H, 2´-H), 4.75 (ddd, *J* = 4.3 Hz, *J* = 10.7 Hz, *J* = 10.7 Hz, 1H, 1-H), 5.08 (s, 2H, OCH_2Ph), 7.10–7.40 (m, 10H, 2 C_6H_5); **10b**: δ = 0.60–2.10 (m, 8H, 2-H to 6-H), 0.85 (d, *J* = 6.4 Hz, 3H, 10-H), 1.00 (s, 9H, $C(CH_3)_3$), 1.14, 1.21 (2s, each 3H, 8-H, 9-H), 2.71 (s, 1H, 2´-H), 4.77 (ddd, *J* = 4.3 Hz, *J* = 10.7 Hz, *J* = 10.7 Hz, 1H, 1-H), 5.11, 5.18 (2d, *J* = 12.4 Hz,

each 1H, OCH_aH_bPh), 7.00–7.40 (m, 10H, 2 C_6H_5). — ^{13}C-NMR ($CDCl_3$): **10a**: δ = 21.74 (q, C-10), 26.12, 26.86 (2q, C-8, C-9), 26.66 (t, C-3), 27.97 (q, C(*C*H_3)$_3$), 31.17 (d, C-5), 33.23 (s, *C*(CH_3)$_3$), 34.47 (t, C-4), 39.73 (s, C-7), 40.71 (t, C-6), 49.94 (d, C-2), 60.79 (d, C-2´), 66.52 (t, OCH_2Ph), 74.88 (d, C-1), 125.09, 125.42, 127.98, 128.25, 128.46 (5d, =*C*-H_{phenyl}), 135.53 (s, =*C*–C_{benzyl}), 151.30 (s, =*C*–C_{phenyl}), 167.04, 168.52 (2s, C=O); **10b**: δ = 21.74 (q, C-10), 26.31 (q, C-8, C-9), 26.76 (t, C-3), 28.07 (q, C(*C*H_3)$_3$), 31.27 (d, C-5), 33.77 (s, *C*(CH_3)$_3$), 34.54 (t, C-4), 39.73 (s, C-7), 41.52 (t, C-6), 50.44 (d, C-2), 60.89 (d, C-2´), 66.49 (t, OCH_2Ph), 75.92 (d, C-1), 124.95, 125.42, 127.88, 128.19, 128.46 (5d, =*C*-H_{phenyl}), 135.67 (s, =*C*–C_{benzyl}), 151.34 (s, =*C*–C_{phenyl}), 167.82, 168.02 (2s, C=O). — MS (GC/MS, ion trap), *m/z* (%): 345 (4), 214 (15), 206 (9), 199 (8), 119 (61), 91 (100). — $C_{30}H_{40}O_4$ (464.7): calcd. C 77.55, H 8.68; found C 77.68, H 8.89.

7. *1-Benzyl 3-[(1R,2S,5R)-2-(2-phenylpropan-2yl)-5-methylcyclohexyl] (2´R/S)-2´-isopropylmalonate* (**11a/b**): 681.7 g (2.93 mmol) (–)-8-phenylmenthol **3** and 1.06 g (4.31 mmol) monobenzyl ester **5** were converted according to the general procedure for the preparation of the menthol esters to afford 1.31 g (2.91 mmol, 99%) **11a/b** as a diastereomeric mixture after flash chromatography (petroleum ether/diethyl ether, 5:1).

FT-IR (film): ν = 1751, 1724 cm^{-1}. — ^{1}H-NMR ($CDCl_3$): δ = 0.60–2.00 (m, 8H, 2-H to 6-H), 0.78 (d, *J* = 6.4 Hz, 3H, 10-H, **11a**), 0.84 (d, *J* = 6.4 Hz, 3H, 10-H, **11b**), 0.89 (d, *J* = 6.7 Hz, in total 6H, CH(C*H*$_3$)$_2$, **11a,** superposed by **11b**), 0.90, 0.91 (2d, *J* = 6.7 Hz, in total 6H, CH(C*H*$_3$)$_2$, **11b**), 1.17, 1.25 (2s, in total 6H, 8-H, 9-H, **11b**), 1.20, 1.28 (2s, in total 6H, 8-H, 9-H, **11a**), 2.20 (dsept, *J* = 6.7 Hz, *J* = 7.6 Hz, 1H, CHC*H*(CH_3)$_2$), 2.51 (d, *J* = 8.1 Hz, 1H, 2´-H, **11a**), 2.74 (d, *J* = 7.6 Hz, 1H, 2´-H, **11b**), 4.79 (ddd, *J* = 4.3 Hz, *J* = 10.7 Hz, *J* = 10.7 Hz, 1H, 1-H), 5.08 (s, 2H, OCH_2Ph, **11a**) 5.15, 5.18 (2d, *J* = 12.2 Hz, each 1H, OCH_aH_bPh, **11b**), 7.10–7.40 (m, 10H, 2 C_6H_5). — ^{13}C-NMR ($CDCl_3$): δ = 19.75, 20.02, 20.39, 20.96 (4q, CH(*C*H_3)$_2$), 21.70 (q, C-10), 25.88, 26.05 (2q, C-8), 26.66, 26.82 (2t, C-3), 26.92 (q, C-9),

28.10, 28.71 (2d, *C*H(CH_3)$_2$), 31.14, 31.24 (2d, C-5), 34.47 (t, C-4), 39.73, 39.83 (2s, C-7), 40.84, 41.45 (2t, C-6), 50.04, 50.35 (2d, C-2), 58.47 (d, C-2´), 66.55, 66.69 (2t, OCH_2Ph), 75.21, 76.09 (2d, C-1), 125.02, 125.49, 127.85, 128.22, 128.45 (5d, =*C*-H_{phenyl}), 135.60 (s, =*C*–C_{benzyl}), 151.14 (s, =*C*–C_{phenyl}), 168.19, 168.52 (2s, C=O). — MS (GC/MS, ion trap), *m/z* (%): 450 (0.5), 359 (0.5), 331 (8), 214 (15), 199 (8), 119 (61), 91 (100). — $C_{29}H_{38}O_4$ (450.6): calcd. C 77.30, H 8.50; found C 77.38, H 8.63.

8. *1-Benzyl 3-{(1R,2S,5R)-2-[2-(4-methoxyphenyl)propan-2-yl)]-5-methylcyclohexyl} (2´R/S)-2´-(tert-butyl)malonate* (**12a/b**): 3.16 g (12.0 mmol) (–)-8-anisylmenthol **4** and 3.00 g (12.0 mmol) monobenzyl ester **6** were converted according to the general procedure for the preparation of the menthol esters to afford 3.61 g (7.30 mmol, 61%) **12a/b** as a diastereomeric mixture after flash chromatography (petroleum ether/diethyl ether, 5:1). An analytical sample of each diastereomer was separated by flash chromatography.

FT-IR (film): ν = 1748, 1728 cm^{-1}. — ^{1}H-NMR ($CDCl_3$): **12a**: δ = 0.65–1.90 (m, 8H, 2-H to 6-H), 0.78 (d, *J* = 6.7 Hz, 3H, 10-H), 1.08 (s, 9H, $C(CH_3)_3$), 1.18, 1.25 (2s, each 3H, 8-H, 9-H), 2.72 (s, 1H, 2´-H), 3.76 (s, 3H, OCH_3), 4.74 (ddd, *J* = 4.3 Hz, *J* = 10.7 Hz, *J* = 10.7 Hz, 1H, 1-H), 5.10 (s, 2H, OCH_2Ph), 6.77–6.83, 7.10–7.17 (2m, 4H, =C-H_{anisyl}), 7.30–7.40 (m, 5H, C_6H_5); **12b**: δ = 0.75–1.60 (m, 8H, 2-H to 6-H), 0.85 (d, *J* = 6.4 Hz, 3H, 10-H), 1.03 (s, 9H, $C(CH_3)_3$), 1.13, 1.21 (2s, each 3H, 8-H, 9-H), 2.81 (s, 1H, 2´-H), 3.71 (s, 3H, OCH_3), 4.74 (ddd, *J* = 4.3 Hz, *J* = 10.7 Hz, *J* = 10.7 Hz, 1H, 1-H), 5.13, 5.17 (2d, *J* = 12.4 Hz, each 1H, OCH_aH_bPh), 6.70–6.81, 7.08–7.15 (2m, 4H, =C-H_{anisyl}), 7.30-7.40 (m, 5H, C_6H_5). — ^{13}C-NMR ($CDCl_3$): **12a**: δ = 21.67 (q, C-10), 26.60 (q, C-8), 26.79 (t, C-3), 26.90 (q, C-9), 28.01 (q, C(*C*H_3)$_3$), 31.18 (d, C-5), 33.26 (s, *C*(CH_3)$_3$), 34.51 (t, C-4), 39.20 (s, C-7), 40.85 (t, C-6), 50.18 (d, C-2), 55.17 (q, OCH_3), 61.03 (d, C-2´), 66.52 (t, OCH_2Ph), 75.05 (d, C-1), 113.33, 126.44 (2d, =*C*-H_{anisyl}), 128.23, 128.46 (2d, =*C*-H_{benzyl}), 135.61 (s, =*C*–C_{benzyl}), 143.26 (s, =*C*–C_{anisyl}), 157.08 (s, =*C*–O_{anisyl}), 167.03, 168.51 (2s, C=O); **12b**: δ = 21.74 (q, C-10), 26.15 (q,

C-8), 26.82 (t, C-3), 26.96 (q, C-9), 28.07 (q, C(*C*H_3)$_3$), 31.27 (d, C-5), 33.84 (s, *C*(CH_3)$_3$), 34.54 (t, C-4), 39.19 (s, C-7), 41.55 (t, C-6), 50.62 (d, C-2), 55.10 (q, OCH_3), 61.03 (d, C-2´), 66.52 (t, OCH_2Ph), 75.99 (d, C-1), 113.19, 126.40 (2d, =*C*-H_{anisyl}), 128.22, 128.46 (2d, =*C*-H_{benzyl}), 135.64 (s, =*C*–C_{benzyl}), 143.35 (s, =*C*–C_{anisyl}), 156.97 (s, =*C*–O_{anisyl}), 167.78, 168.02 (2s, C=O). — MS (GC/MS), *m/z* (%): 494 (4), 345 (0.5), 245 (1), 149 (100), 91 (17), 84 (17). — $C_{31}H_{42}O_5$ (494.7): calcd. C 75.27, H 8.56; found C 75.16, H 8.60.

9. 2´-*[(1R,2S,5R)-2-Isopropyl-5-methylcyclohexyloxycarbonyl] (2´R/S)-3´-methylbutanoic acid* (**13a/b**):

785.9 mg (2.10 mmol) benzyl ester **7a/b** were converted according to the general procedure for the hydrogenation of the benzyl esters to afford 590.7 mg (2.08 mmol, 99%) of the free carboxylic acid **13a/b** as a diastereomeric mixture.

FT-IR (film): ν = 3600–2600, 1737, 1713 cm^{-1}. — ^{1}H-NMR ($CDCl_3$): δ = 0.75 (d, *J* = 6.9 Hz, 3H, 10-H), 0.80-2.10 (m, 9H, 2-H to 7-H), 1.02-1.06 (m, 6H, CH(C*H*$_3$)$_2$), 0.88, 0.90 (2d, *J* = 7.2 Hz, in total 6H, 8-H, 9-H), 2.39 (dsept, 1H, CHC*H*(CH_3)$_2$), 3.15 (d, *J* = 8.1 Hz, 1H, 2´-H), 4.72, 4.74 (2ddd, *J* = 4.8 Hz, *J* = 10.9 Hz, *J* = 10.9 Hz, 1H, 1-H). — ^{13}C-NMR ($CDCl_3$): δ = 15.94, 16.07 (2q, C-10), 20.25, 20.35 (2q, CH(*C*H_3)$_2$), 20.69, 21.94 (2q, C-8, C-9), 23.18, 23.29 (2t, C-3), 26.08 (d, C-5), 29.05, 29.28 (2d, *C*H(CH_3)$_2$), 31.41 (d, C-7), 34.17 (t, C-4), 40.40, 40.64 (2t, C-6), 46.81, 46.91 (2d, C-2), 58.57, 58.94 (2d, C-2´), 75.92, 75.99 (d, C-1), 168.79, 169.20 (2s, C=O_{ester}), 173.27, 173.44 (2s, C=O_{acid}). — MS (GC/MS, **13** as methylester), *m/z* (%): 283 (0.1), 267 (0.4), 255 (0.2), 223 (0.3), 161 (25), 143 (55), 138 (100), 123 (36), 95 (88), 83 (55), 81 (66). IR and ^{1}H-NMR are identical with those reported for the compound by *Fukumoto*[SI-1].

10. *2´-[(1R,2S,5R)-2-Isopropyl-5-methylcyclohexyloxycarbonyl)-(2´R/S)-3´,3´-dimethylbutanoic acid* (**14a/b**): 1.62 g (4.17 mmol benzyl ester **8a/b** were converted

according to the general procedure for the hydrogenation of the benzyl esters to afford 1.20 g (4.02 mmol, 96%) of the free carboxylic acid **14a/b** as a diastereomeric mixture.

FT-IR (film): ν = 3600–2600, 1753, 1712 cm^{-1}. — ^{1}H-NMR ($CDCl_3$): δ = 0.74 (d, *J* = 6.9 Hz, 3H, 10-H), 0.80–2.10 (m, 9H, 2-H to 7-H), 1.14 (s, 9H, $C(CH_3)_3$), 0.85–0.92 (m, in total 6H, 8-H, 9-H), 3.24 (s, 1H, 2´-H), 4.72, 4.77 (2ddd, *J* = 4.5 Hz, *J* = 10.7 Hz, *J* = 10.7 Hz, 1H, 1-H). — ^{13}C-NMR ($CDCl_3$): δ = 15.80, 16.07 (2q, C-10), 20.76, 21.94 (2q, C-8, C-9), 23.08, 23.32 (2t, C-3), 25.98, 26.12 (2d, C-5), 28.14 (q, $C(CH_3)_3$), 31.41 (d, C-7), 33.93, 34.34 (2s, $C(CH_3)_3$), 34.17 (t, C-4), 40.44, 40.71 (2t, C-6), 46.74, 46.94 (2d, C-2), 61.09, 61.60 (2d, C-2´), 76.09, 76.19 (2d, C-1), 169.06, 170.15 (2s, $C{=}O_{ester}$), 171.89, 172.63 (2s, $C{=}O_{acid}$). — MS (GC/MS, **14** as methylester), *m/z* (%): 297 (0.5), 281 (0.6), 256 (1), 175 (24), 157 (20), 138 (100), 123 (18), 118 (10), 101 (42), 95 (37), 83 (36), 69 (14), 57 (29). — $C_{17}H_{30}O_4$ (298.4): calcd. C 68.42, H 10.13; found C 68.23, H 10.32.

11. 2´-*[(1R,2S,5R)-2-(tert-Butyl)-5-methylcyclohexyloxycarbonyl] (2´R/S)-3´,3´-dimethylbutanoic acid* (**15a/b**): 841 mg (2.11 mmol) benzyl ester **9a/b** were converted according to the general procedure for the hydrogenation of the benzyl esters to afford 653 mg (2.09 mmol, 99%) of the free carboxylic acid **15a/b** as a diastereomeric mixture.

FT-IR (film): ν = 3600–2800, 1749, 1711 cm^{-1}. — ^{1}H-NMR ($CDCl_3$): δ = 0.87 (d, *J* = 5.1 Hz, 3H, 11-H), 0.8–2.05 (m, 8H, 2-H to 6-H), 0.89 (s, 9H, 8-H to 10-H), 1.14, 1.16 (2s, 9H, $COCHC(CH_3)_3$), 3.20 (s, 1H, 2´-H), 4.79 (ddd, *J* = 4.3 Hz, *J* = 10.5 Hz, *J* = 10.5 Hz, 1H, 1-H, **15a**), 4.84 (ddd, *J* = 4.3 Hz, *J* = 10.5 Hz, *J* = 10.5 Hz, 1H, 1-H, **15b**, superposed by **15a**). — ^{13}C-NMR ($CDCl_3$): δ = 21.80 (q, C-11), 26.55, 26.51 (2t, C-3), 28.17 (q, $C(CH_3)_3$), 28.98 (q, C-8 to C-10), 31.31 (d, C-5), 32.55 (s, C-7), 33.80 (s, $C(CH_3)_3$), 34.54, 34.64 (2t, C-4), 41.01, 41.65 (2t, C-6), 49.70, 49.87 (2d, C-2), 61.97 (d, C-2´), 76.63, 77.24 (2d, C-1), 167.38, 168.32 (2s, C=O). — MS (GC/MS, **15** as methylester), *m/z* (%): 311 (1), 271 (2), 269 (1), 175 (100),

157 (77), 152 (14), 119 (18), 101 (50), 57 (52). — HR MS (NH_3-DCI): $C_{18}H_{32}O_4$: calcd. 330.2644; found 330.2632{M +NH_4^+}.

12. *3´,3´-Dimethyl-2´-[(1R,2S,5R)-5-methyl-2-(2-phenylpropan-2-yl)cyclohexyloxycarbonyl] (2´R/S)-butanoic acid* (**16a/b**): 631 mg (1.36 mmol) benzyl ester **10a/b** were converted according to the general procedure for the hydrogenation of the benzyl esters to afford 441.5 mg (1.18 mmol, 87%) of the free carboxylic acid **16a/b** as a diastereomeric mixture. An analytical sample of **16a** was separated by flash chromatography (petroleum ether/diethyl ether, 1:1).

FT-IR (film): ν = 3600–2800, 1740, 1711 cm^{-1}. — ^{1}H-NMR ($CDCl_3$): **16a**: δ = 0.70–2.00 (m, 8H, 2-H to 6-H), 0.85 (d, *J* = 6.4 Hz, 3H, 10-H), 1.07 (s, 9H, $C(CH_3)_3$), 1.20, 1.30 (2s, each 3H, 8-H, 9-H), 2.59 (s, 1H, 2´-H), 4.79 (ddd, *J* = 4.3 Hz, *J* = 10.7 Hz, *J* = 10.7 Hz, 1H, 1-H), 7.10–7.40 (m, 5H, C_6H_5). — ^{13}C-NMR ($CDCl_3$): **16a**: δ = 21.70 (q, C-10), 26.25, 26.75 (2q, C-8, C-9), 26.79 (t, C-3), 28.00 (q, $C(CH_3)_3$), 31.27 (d, C-5), 33.29 (s, $C(CH_3)_3$), 34.51 (t, C-4), 39.73 (s, C-7), 40.74 (t, C-6), 50.01 (d, C-2), 60.83 (d, C-2´), 75.62 (d, C-1), 125.12, 125.42, 128.02 (3d, =*C*-H_{phenyl}), 151.24 (s, =*C*–C_{phenyl}), 166.94 (s, C=O_{ester}), 174.49 (s, C=O_{acid}). — MS (GC/MS, **16** as methylester), *m/z* (%): 388 (0.3), 269 (5), 214 (15), 199 (3), 175 (4), 119 (61), 105 (14), 91 (100), 57 (10). — HR MS (NH_3-DCI): $C_{23}H_{34}O_4$: calcd. 392.28008; found 392.2793 {M +NH_4^+}.

13. *3´-Methyl-2´-[(1R,2S,5R)-2-(1-Methyl-1-phenylethyl)-5-methylcyclohexyloxycarbonyl)] (2´R/S)-butanoic acid* (**17a/b**): 1.135 g (2.52 mmol) benzyl ester **11a/b** was converted according to the general procedure for the hydrogenation of the benzyl esters to afford 904 mg (2.51 mmol, 99%) of the free carboxylic acid **17a/b** as a diastereomeric mixture.

FT-IR (film): ν = 3600–2800, 1735, 1711 cm^{-1}. — ^{1}H-NMR ($CDCl_3$): δ = 0.70–2.00 (m, 8H, 2-H to 6-H), 0.85 (d, *J* = 6.4 Hz, 3H, 10-H, **17a**), 0.87 (d, *J* = 6.4 Hz, 3H, 10-H, **17b**), 0.88,

0.90 (2d, *J* = 6.7 Hz, in total 6H, CH(C*H*$_3$)$_2$, **17b**), 0.97, 1.00 (2d, *J* = 6.7 Hz, in total 6H, CH(C*H*$_3$)$_2$, **17a**), 1.20, 1.21, 1.30 (3s, in total 6H, 8-H, 9-H), 2.34 (d, *J* = 6.2 Hz, 1H, 2´-H, **17b**), 2.47 (d, *J* = 7.6 Hz, 1H, 2´-H, **17a**), 4.84 (ddd, *J* = 4.3 Hz, *J* = 10.7 Hz, *J* = 10.7 Hz, 1H, 1-H, **17a**, superposed by **17b**), 4.88 (ddd, *J* = 4.3 Hz, *J* = 10.7 Hz, *J* = 10.7 Hz, 1H, 1-H, **17b**), 7.10–7.30 (m, 5H, C_6H_5). — ^{13}C-NMR ($CDCl_3$): δ = 19.68, 19.85, 20.19, 20.86 (4q, CH(*C*H$_3$)$_2$), 21.74 (q, C-10), 25.81, 27.13 (2q, C-8, C-9), 26.42, 26.69 (2t, C-3), 28.21, 28.41 (2d, *C*H(CH$_3$)$_2$), 30.19, 31.27 (2d, C-5), 34.41 (t, C-4), 39.52, 39.81 (2s, C-7), 40.81, 41.48 (2t, C-6), 49.97, 50.31 (2d, C-2), 56.55, 58.03 (2d, C-2´), 75.69, 76.39 (2d, C-1), 125.12 125.36, 125.42, 127.95, 128.05 (5d, =*C*-H$_{phenyl}$), 151.24 (s, =*C*–C$_{phenyl}$), 167.68 (s, C=O$_{ester}$), 170.98, 171.69 (s, C=O$_{acid}$). — MS (GC/MS, **17** as methylester), *m/z* (%): 374 (1), 343 (0.5), 255 (10), 214 (44), 199 (4), 143 (21), 119 (96), 118 (100), 105 (15), 91 (26). IR and ^{1}H-NMR are identical with those reported for the compound by *Fukumoto*[SI-1].

14. *2´-(1R,2S,5R)-2-[2-(4-Methoxyphenyl)propan-2-yl)-5-methylcyclohexyloxycarbonyl] (2´R/S)-3´,3´-dimethylbutanoic acid* (**18a/b**): 2.56 g (5.12 mmol) benzyl ester **12a/b** were converted according to the general procedure for the hydrogenation of the benzyl esters to afford 2.02 g (5.00 mmol, 98%) of the free carboxylic acid **18a/b** as a diastereomeric mixture. FT-IR (film): ν = 3600–2600, 1747, 1709 cm^{-1}. — ^{1}H-NMR ($CDCl_3$): δ = 0.65–1.90 (m, 8H, 2-H to 6-H), 0.85, 0.90 (2d, *J* = 6.4 Hz, in total 3H, 10-H), 0.97, 1.12 (2s, in total 9H, C(CH$_3$)$_3$), 1.20, 1.21, 1.26, 1.29 (4s, in total 6H, 8-H, 9-H), 2.31, 2.72 (2s, in total 1H, 2´-H), 3.79, 3.81 (2s, in total 3H, OCH$_3$), 4.78, 4.85 (2ddd, *J* = 4.3 Hz, *J* = 10.7 Hz, *J* = 10.7 Hz, in total 1H, 1-H), 6.77–6.88, 7.10–7.20 (2m, 4H, =C-*H*$_{anisyl}$). — ^{13}C-NMR ($CDCl_3$): δ = 21.74, 23.89 (2q, C-10), 26.55, 26.96 (2q, C-8, C-9), 26.89 (t, C-3), 28.07, 28.31 (2q, C(*C*H$_3$)$_3$), 29.18, 31.31 (2d, C-5), 33.39 (s, *C*(CH$_3$)$_3$), 34.54 (t, C-4), 39.22 (s, C-7), 40.84, 41.48 (2t, C-6), 50.18, 50.45 (2d, C-2), 55.23 (q, OCH$_3$), 60.25, 60.96 (2d, C-2´), 75.82, 77.00 (2d, C-1),

113.39, 113.56, 125.93, 126.47 (4d, =*C*-H_{anisyl}), 143.28 (s, =*C*–C_{anisyl}), 157.13 (s, =*C*–O_{anisyl}), 167.11 (s, C=O_{ester}), 173.85 (s, C=O_{acid}). — MS (GC/MS, **18** as methylester), *m/z* (%): 418 (8), 269 (1), 245 (6), 229 (3), 173 (2), 149 (100), 135 (18), 121 (17), 109 (8), 91 (17), 84 (17), 57 (10). — HR MS (GC-EI, **18a/b** as trimethylsilylester): $C_{24}H_{35}O_5SiMe_3$: calcd. 476.2958; found 476.2924.

15. *(1R,2S,5R)-2-Isopropyl-5-methylcyclohexyl (2´R/S)-2´-isopropyl-4,4-dimethylpentanoate* (**21a/b**): 74.5 mg (0.262 mmol) of **14a/b** and 364.5 mg (3.14 mmol) *tert*-butylacetic acid were electrolyzed according to the general procedure for the Kolbe electrolysis to afford 41.8 mg (0.135 mmol, 51%) **21a/b** as a diastereomeric mixture after flash chromatography and HPLC (petroleum ether/diethyl ether, 20:1). The diasteromers **21a** and **21b** have not been separated. The diasteromeric ratio was determined by inverse gated coupling ^{13}C-NMR to be 52.5 : 47.5 (5% *de*).

FT-IR (film): ν = 1730 cm^{-1}. — ^{1}H-NMR ($CDCl_3$): δ = 0.71, 0.73 (2d, *J* = 6.8 Hz, 3H, 10-H), 0.85, 0.86 (2s, 9H, C(C*H*$_3$)$_3$), 0.80–2.20 (m, 13H, 2-H to 7-H, 2´-H, C*H*(CH_3)$_2$), 4.62, 4.64 (2ddd, *J* = 10.7 Hz, *J* = 10.7 Hz, *J* = 4.3 Hz, 1H, 1-H). — ^{13}C-NMR ($CDCl_3$): δ = 15.60, 15.67 (2q, C-8), 19.55, 19.88, 20.19, 20.35 (4q, COCHCH(*C*H_3)$_2$), 20.96 (q, C-9), 22.07 (q, C-10), 22.84, 22.98 (2t, C-3), 25.68 (d, C-7), 29.32, 29.42 (2q, C(*C*H_3)$_3$), 30.50, 30.56 (2s, *C*(CH_3)$_3$), 31.41 (d, C-5), 31.84, 32.15 (2d, *C*H(CH_3)$_2$), 34.34 (t, C-4), 40.51, 40.88 (2t, C-6), 41.92, 42.16 (2t, *C*H_2*t*Bu), 47.01 (d, C-2), 47.99, 48.46 (2d, C-2´), 74.00, 74.07 (2d, C-1), 176.11, 176.34 (2s, C=O). — MS (GC/MS), *m/z* (%): 309 (0.2), 295 (0.4), 268 (0.6), 173 (14), 157 (26), 155 (12), 138 (100), 127 (10), 95 (41), 83 (54), 71 (45), 57 (56). — $C_{20}H_{38}O_2$ (310.5): calcd. C 77.36, H 12.33; found C 77.42, H 12.40.

16. *(1R,2S,5R)-2-Isopropyl-5-methylcyclohexyl (2´R/S)-2´-(tert-butyl)-4´,4´-dimethylpentanoate* (**22a/b**): 98.0 mg (0.262 mmol) of **13a/b** and 384.0 mg (3.31 mmol) *tert*-butylacetic acid were electrolyzed according to the general procedure for the Kolbe electrolysis to afford 62.0 mg (0.191 mmol, 58%) **22a/b** as a diastereomeric mixture after

flash chromatography and HPLC (petroleum ether/diethyl ether, 50:1). The diasteromers **22a** and **22b** have not been separated. The diasteromeric ratio was determined by GLC to be 55 : 45 (10% *de*).

FT-IR (film): ν = 1726 cm^{-1}. — ^{1}H-NMR ($CDCl_3$): δ = 0.68, 0.72 (2d, *J* = 6.9 Hz, 3H, 10-H), 0.86 (d, *J* = 3.6 Hz, 6H, CH(C*H*$_3$)$_2$), 0.94 (s, 18H, 2 $C(CH_3)_3$), 0.80–2.20 (m, 12H, 2-H to 7-H, *t*BuCHCH_2), 4.57, 4.61 (2ddd, *J* = 10.9 Hz, *J* = 10.8 Hz, *J* = 4.3 Hz, 1H, 1-H). — ^{13}C-NMR ($CDCl_3$): δ = 15.47 (q, C-10), 21.03, 22.07 (2q, C-8, C-9), 22.71 (t, C-3), 25.41 (d, C-7), 27.67, 27.80 (2q, C(*C*H$_3$)$_3$), 29.28, 29.42 (2q, C(*C*H$_3$)$_3$), 30.46, 30.63 (2s, *C*(CH$_3$)$_3$), 31.37, 31.44 (2d, C-5), 33.46, 33.73 (2s, *C*(CH$_3$)$_3$), 34.34 (t, C-4), 40.34, 40.88 (2t, C-6), 41.38, 42.02 (2t, *t*BuCH*C*H$_2$), 46.94, 47.01 (d, C-2), 51.42, 52.10 (2d, C-2´), 74.44 (d, C-1), 176.11, 176.34 (2s, C=O). — MS (GC/MS), *m/z* (%): 309 (0.5), 268 (3), 187 (10), 171 (22), 169 (10), 138 (100), 127 (18), 95 (24), 85 (16), 83 (45), 57 (51). — $C_{21}H_{40}O_2$ (324.6): calcd. C 77.72, H 12.42; found C 77.54, H 12.50.

17. *(1R,2S,5R)-2-(tert-Butyl)-5-methylcyclohexyl (2´R)-2´-(tert-butyl)-4´,4´-dimethyl-pentanoate* (**23a**) and *(1R,2S,5R)-2-(tert-butyl)-5-methylcyclohexyl (2´S)-2´-(tert-butyl)-4´,4´-dimethylpentanoate* (**23b**): 128.2 mg (0.410 mmol) of **15a/b** and 473.0 mg (4.072 mmol) *tert*-butylacetic acid were electrolyzed according to the general procedure for the Kolbe electrolysis to afford 59.7 mg (0.176 mmol, 43%) **23a** and 36.7 mg (0.108 mmol, 26%) **23b** after flash chromatography and HPLC (methylene chloride/petroleum ether, 1:3). The diastereomeric ratio was determined by GLC to be **23a**:**23b** = 63.5: 36.5 (27% *de*).

m.p. 49–53°C (**23b**). — $[\alpha]_D^{20}$ = –101.6° (**23a**, *c* = 0.6 in trichloromethane); –18.5° (**23b**, *c* = 1.0 in trichloromethane). — FT-IR (film): ν = 1723 cm^{-1}. — ^{1}H-NMR ($CDCl_3$): **23a**: δ = 0.86 (d, *J* = 6.4 Hz, 3H, 11-H), 0.88, 0.95, 0.96 (3s, each 9H, *t*Bu), 0.80–1.70 (m, 6H), 1.37 (dd, *J* = 1 Hz, *J* = 14.3 Hz, 1H, *t*BuCH*CH$_a$*H$_b$), 1.70 (dd, *J* = 14.3 Hz, *J* = 9.3 Hz, 1H,

*t*BuCH*C*H$_a$*H$_b$*), 1.86 (m$_c$, 1H), 2.13 (dd, *J* = 1 Hz, *J* = 9.3 Hz, 1H, 2´-C), 2.19 (m$_c$, 1H), 4.61 (ddd, *J* = 10.5 Hz, *J* = 10.4 Hz, *J* = 3.8 Hz, 1H, 1-H); **23b**: δ = 0.88 (d, *J* = 6.5 Hz, 3H, 11-H), 0.86, 0.93, 0.94 (3s, each 9H, C(CH_3)$_3$), 0.80–1.60 (m, 5H), 1.29 (dd, *J* = 0.7 Hz, *J* = 14.1 Hz, 1H, *t*BuCH*CH$_a$*H$_b$), 1.66, 1.86 (2m$_c$, each 1H), 1.89 (dd, *J* = 14.1 Hz, *J* = 9.8 Hz, 1H, *t*BuCH*C*H$_a$*H$_b$*), 2.06 (dd, *J* = 0.7 Hz, *J* = 9.8 Hz, 1H, 2´-H), 2.22 (m$_c$, 1H), 4.58 (ddd, *J* = 10.5 Hz, *J* = 10.4 Hz, *J* = 3.8 Hz, 1H, 1-H). — ^{13}C-NMR ($CDCl_3$): **23a**: δ = 21.90 (q, C-11), 27.79 (t, C-3), 27.87, 29.18, 29.59 (3q, C(*C*H$_3$)$_3$), 30.80 (s, *C*(CH$_3$)$_3$), 31.31 (d, C-5), 32.55 (s, C-7), 33.93 (s, *C*(CH$_3$)$_3$), 34.88 (t, C-4), 41.08 (t, C-6), 41.82 (t, *t*BuCH*C*H$_2$), 50.11 (d, C-2), 52.37 (d, C-2´), 77.03 (d, C-1), 176.48 (s, C=O); **23b**: δ = 21.94 (q, C-11), 26.62 (t, C-3), 28.24, 29.05, 29.62 (3q, C(*C*H$_3$)$_3$), 30.33 (s, *C*(CH$_3$)$_3$), 31.34 (d, C-5), 32.69 (s, C-7), 33.97 (s, *C*(CH$_3$)$_3$), 34.91 (t, C-4), 40.94 (t, C-6), 41.45 (t, *t*BuCH*C*H$_2$), 50.24 (d, C-2), 51.46 (d, C-2´), 77.10 (d, C-1), 175.09 (s, C=O). — MS (GC/MS), *m/z* (%): 323 (2), 282 (0.5), 187 (61), 169 (12), 153 (36), 152 (34), 131 (10), 97 (40), 94 (28), 57 (100). — $C_{22}H_{42}O_2$ (338.6): calcd. C 78.05, H 12.50; found C 78.11, H 12.74.

18.*(1R,2S,5R)-5-Methyl-2-(1-methyl-1-phenylethyl)cyclohexyl (2´R)-2´-isopropyl-4´,4´-dimethylpentanoate* (**24a**) and *(1R,2S,5R)-5-methyl-2-(1-methyl-1-phenylethyl)cyclohexyl (2´S)-2´-isopropyl-4´,4´-dimethylpentanoate* (**24b**): 142.2 mg (0.394 mmol) of **17a/b** and 384.0 mg (3.31 mmol) *tert*-butylacetic acid were electrolyzed according to the general procedure for the Kolbe electrolysis to afford 32.9 mg (0.085 mmol, 22%) **24a/b** as a diastereomeric mixture after flash chromatography (petroleum ether/diethyl ether, 20:1). The diasteromers **24a** and **24b** have not been separated. The diasteromeric ratio was determined by GLC to be **24a**:**24b** = 69 : 31 (38% *de*).

FT-IR (film): ν = 1725 cm^{-1}. — ^{1}H-NMR ($CDCl_3$): δ = 0.65, 0.77 (2d, 6H, CH(C*H$_3$*)$_2$), 0.81, 0.83 (2d, *J* = 5.5 Hz, 3H, 10-H), 0.76, 0.75 (2s, 9H, C(CH_3)$_3$), 0.80–2.05 (m, 12H), 1.13,

1.16, 1.27, 1.31 (4s, 6H, 8-H, 9-H), 4.60, 4.64 (2ddd, *J* = 9.1 Hz, *J* = 8.7 Hz, *J* = 3.6 Hz, 1H, 1-H), 7.02–7.30 (m, 5H, C_6H_5). — ^{13}C-NMR ($CDCl_3$): δ = 18.12, 18.66, 20.12, 21.10 (4q, CH(*C*H_3)$_2$), 21.82, 21.85 (2q, C-10), 25.15, 26.28, 26.72, 28.49 (4q, C-8, C-9), 26.95, 27.31 (t, C-3), 29.43 (q, C(*C*H_3)$_3$), 30.24 (d, *C*H(CH_3)$_2$), 30.31, 30.49 (2s, *C*(CH_3)$_3$), 31.30, 31.54 (2d, C-5), 34.68, 34.78 (2t, C-4), 39.85, 40.11 (2s, C-7), 40.50 (t, C-6), 41.15, 41.35 (2t, *t*Bu*C*H_2), 46.51 (d, C-2´, **24b**), 47.33 (d, C-2´, **24a**), 50.09, 50.28 (2d, C-2), 75.32, 75.55 (2d, C-1), 124.96, 125.12, 125.41, 125.58, 127.90, 127.93 (6d, =*C*-H_{phenyl}), 151.83, 151.29 (2s, =*C*–C_{phenyl}), 175.11 (s, C=O, **24b**), 176.40 (s, C=O, **24a**). — MS (GC/MS), *m/z* (%): 386 (0.5), 267 (14), 215 (7), 214 (17), 199 (4), 173 (12), 119 (100), 118 (64), 105 (14), 91 (10), 57 (8). — HR MS (NH_3-DCI): $C_{26}H_{42}O_2$: calcd. 404.3529; found 404.3520 {M +NH_4^+}.

19. *(1R,2S,5R)-5-Methyl-2-(1-methyl-1-phenylethyl)cyclohexyl (2´R)-2´-(tert-butyl)-4´,4´-dimethylpentanoate* (**25a**) and *(1R,2S,5R)-5-methyl-2-(1-methyl-1-phenylethyl)cyclohexyl (2´S)-2´-(tert-butyl)-4´,4´-dimethylpentanoate* (**25b**): 148.3 mg (0.396 mmol) of **16a/b** and 463.9 mg (3.99 mmol) *tert*-butylacetic acid were electrolyzed according to the general procedure for the Kolbe electrolysis to afford 49.1 mg (0.123 mmol, 31%) **25a** and 14.1 mg (0.035 mmol, 8.9%) **25b** after flash chromatography and HPLC (methylene chloride/petroleum ether, 1:4). The diastereomeric ratio was determined by GLC to be **25a** : **25b** = 82.5 : 17.5 (65% *de*).

$[\alpha]_D^{20}$ = –34.1° (**25a**, *c* = 0.8 in trichloromethane); –6.8° (**25b**, *c* = 1.8 in trichloromethane). — FT-IR (film): ν = 1720 cm^{-1}. — ^{1}H-NMR ($CDCl_3$): **25a**: δ = 0.86 (d, *J* = 6.2 Hz, 3H, 10-H), 0.85, 0.94 (2s, each 9H, C(C*H_3*)$_3$), 1.24, 1.46 (2s, each 3H, 8-H, 9-H), 0.80–1.65 (m, 8H), 2.00–2.10 (m, 1H), 1.90, 2.29 (2m_c, each 1H), 4.65 (ddd, *J* = 10.5 Hz, *J* = 10.4 Hz, *J* = 3.6 Hz, 1H, 1-H), 7.12–7.71 (m, 5H, C_6H_5); **25b**: δ = 0.88 (d, *J* = 5.5 Hz, 3H, 10-H), 0.84 (s, 18H, C(C*H_3*)$_3$), 1.19, 1.41 (2s, each 3H, 8-H, 9-H), 0.80–1.65 (m, 6H), 1.80–2.40 (m, 5H),

4.57 (ddd, J = 10.5 Hz, J = 10.4 Hz, J = 3.6 Hz, 1H, 1-H), 7.12–7.35 (m, 5H, C_6H_5). — ^{13}C-NMR ($CDCl_3$): **25a**: δ = 21.84 (q, C-10), 24.47, 28.95 (2q, C-8, C-9), 27.57 (t, C-3), 27.73, 29.62 (2q, C(CH$_3$)$_3$), 30.80 (s, C(CH$_3$)$_3$), 31.44 (d, C-5), 33.87 (s, C(CH$_3$)$_3$), 34.81 (t, C-4), 40.17 (s, C-7), 41.01 (t, C-6), 41.95 (t, *t*BuCH*C*H$_2$), 50.48 (d, C-2), 51.69 (d, C-2´), 76.80 (d, C-1), 125.12, 125.63, 127.98 (3d, C-2´ to C-4´), 151.51 (s, C-1´), 176.68 (s, C=O); **25b**: δ = 21.87 (q, C-10), 25.37, 27.57 (2q, C-8, C-9), 27.46 (t, C-3), 28.24, 29.52 (2q, C(CH$_3$)$_3$), 30.33 (s, C(CH$_3$)$_3$), 31.47 (d, C-5), 33.70 (s, C(CH$_3$)$_3$), 34.95 (t, C-4), 40.03 (s, C-7), 40.61 (t, C-6), 41.35 (t, *t*BuCH*C*H$_2$), 50.65 (d, C-2), 50.85 (d, C-2´), 77.00 (d, C-1), 124.95, 125.46, 127.95 (3d, =C-H$_{phenyl}$), 151.94 (s, =C–C$_{phenyl}$), 175.06 (s, C=O). — MS (GC/MS), *m/z* (%): 400 (0.1), 281 (15), 215 (16), 214 (22), 199 (6), 187 (12), 171 (11), 119 (98), 118 (100), 105 (46), 91 (29), 57 (59). — HR MS (NH_3-DCI): $C_{27}H_{44}O_2$: calcd. 418.3685; found 418.3687{M +NH_4^+}.

20. *Di[(1R,2S,5R)-2-isopropyl-5-methylcyclohexyl] (2´R*,3´R*)-2´,3´-diisopropylsuccinate* (**26a**), *di[(1R,2S,5R)-2-isopropyl-5-methylcyclohexyl] (2´R,3´S)-2´,3´-diisopropylsuccinate* (**26b**) and *di[(1R,2S,5R)-2-isopropyl-5-methylcyclohexyl] (2´S*,3´S*)-2´,3´-diisopropyl-succinate* (**26c**): 469.0 mg (1.89 mmol) of **14a/b** were electrolyzed according to the general procedure for the Kolbe electrolysis to afford 51.7 mg (0.108 mmol, 13%) **26a**, 88.9 mg (0.186 mmol, 23%) **26b** and 35.9 mg (0.075 mmol, 9%) **26c** after flash chromatography and HPLC (petroleum ether/diethyl ether, 40:1). The diastereomeric ratio was determined by GLC to be **26a** : **26b** : **26c** = 1.17 : 2.00 : 0.81.

m.p. 136–137 °C (**26a**); 145–146 °C (**26b**); 108–109 °C (**26c**). — $[\alpha]_D^{20}$ = –43.5° (**26a**, *c* = 0.8 in trichloromethane); –65.2° (**26b**, *c* = 1.2 in trichloromethane); –102.8° (**26c**, *c* = 1.0 in trichloromethane). — FT-IR (KBr): ν = 1725 cm^{-1}. — ^{1}H-NMR ($CDCl_3$): **26a**: δ = 0.73 (d, J = 6.9 Hz, 6H, 2× 10-H), 0.87–0.91, 1.04 (superposing doublets, 24H, 2× 8-H, 2× 9-H,

2× $CH(CH_3)_2$), 0.80–1.10 (m, 6H), 1.30–1.55 (m, 4H), 1.66 (m_c, 4H), 1.90–2.10 (m, 6H), 2.53 (m_c, 2H, 2´-H, 3´-H), 4.66 (ddd, *J* = 10.9 Hz, *J* = 10.8 Hz, *J* = 4.3 Hz, 2H, 2× 1-H); **26b**: δ = 0.73, 0.74 (2d, *J* = 6.9 Hz, 6H, 2× 10-H), 0.87–0.99 (superposing doublets, 24H, 2× 8-H, 2× 9-H, 2× $CH(CH_3)_2$), 0.80–1.10 (m, 6H), 1.30–2.10 (m, 14H), 2.81 (m_c, 2H, 2´-H, 3´-H), 4.64, 4.70 (2ddd, *J* = 10.9 Hz, *J* = 10.8 Hz, *J* = 4.3 Hz, 2H, 2× 1-H); **26c**: δ = 0.73 (d, *J* = 6.9 Hz, 6H, 2× 10-H), 0.86 (d, *J* = 6.9 Hz, 6H, 1× $CH(CH_3)_2$), 0.89, 0.90 (2d, *J* = 7.0 Hz, each 6H, 8-H, 9-H), 1.08 (d, *J* = 6.9 Hz, 6H, 1× $CH(CH_3)_2$), 0.8–1.1 (m, 6H), 1.30–1.55 (m, 4H), 1.66 (m_c, 4H), 1.90–2.10 (m, 6H), 2.67 (m_c, 2H, 2´-H, 3´-H), 4.60 (ddd, *J* = 10.9 Hz, *J* = 10.8 Hz, *J* = 4.3 Hz, 2H, 2× 1-H). — ^{13}C-NMR ($CDCl_3$): **26a**: δ = 15.74 (q, C-10), 19.51, 20.86 (2q, C-8, C-9), 21.33, 22.11 (2q, $CH(CH_3)_2$), 22.98 (t, C-3), 25.81 (d, C-7), 27.23 (d, $CH(CH_3)_2$), 31.47 (d, C-5), 34.37 (t, C-4), 40.74 (t, C-6), 46.81 (d, C-2), 51.83 (d, C-2´, C-3´), 74.47 (d, C-1), 173.11 (s, C=O); **26b**: δ = 15.47, 15.57 (2q, C-10), 16.85, 17.05, 20.86, 20.93 (4q, C-8, C-9), 21.90, 22.04 (2q, $CH(CH_3)_2$), 22.75, 22.88 (2t, C-3), 25.64, 25.78 (2d, C-7), 28.88, 29.22 (2d, $CH(CH_3)_2$), 31.40, 31.47 (2d, C-5), 34.27 (t, C-4), 40.74, 41.15 (2t, C-6), 46.81, 46.87 (2d, C-2), 51.35, 51.56 (2d, C-2´, C-3´), 74.44, 74.61 (2d, C-1), 172.40, 172.70 (s, C=O); **26c**: δ = 16.11 (q, C-10), 18.00, 20.86 (2q, C-8, C-9), 22.07 (q, $CH(CH_3)_2$), 23.25 (t, C-3), 25.68 (d, C-7), 26.68 (d, $CH(CH_3)_2$), 31.44 (d, C-5), 34.34 (t, C-4), 41.18 (t, C-6), 46.94 (d, C-2), 50.45 (d, C-2´, C-3´), 74.34 (d, C-1), 173.21 (s, C=O). — MS (GC/MS), *m/z* (%): 478 (1.5), 393 (1), 341 (4), 325 (1), 239 (18), 203 (24), 185 (52), 157 (6), 139 (100), 101 (46), 83 (57). — $C_{30}H_{54}O_4$ (478.8): calcd. C 75.26, H 11.37; found C 75.23, H 11.27.

21. *Di[(1R,2S,5R)-2-isopropyl-5-methylcyclohexyl] (2´R*,3´R*)-2´,3´- di(tert-butyl)succinate* (**27a**), *di[(1R,2S,5R)-2-isopropyl-5-methylcyclohexyl] (2´R,3´S)-2´,3´- di(tert-butyl)succinate* (**27b**) and *di[(1R,2S,5R)-2-isopropyl-5-methylcyclohexyl] (2´S*,3´S*)-2´,3´- di(tert-butyl)-succinate* (**27c**): 546.9 mg (1.83 mmol) of **13a/b** were electrolyzed according to the general

procedure for the Kolbe electrolysis to afford 80.1 mg (0.158 mmol, 17%) **27a**, 118.1 mg (0.233 mmol, 26%) **27b** and 31.7 mg (0.063 mmol, 7%) **27c** after flash chromatography and HPLC (methylene chloride/petroleum ether, 1:2). The diastereomeric ratio was determined by GLC to be **27a** : **27b** : **27c** = 1.39 : 2.00 : 0.67.

m.p. 40–45 °C (**27a**); 177–182 °C (**27b**); 145–150 °C (**27c**). — $[\alpha]_D^{20}$ = –89.8° (**27a**, *c* = 0.9 in trichloromethane); –39.7° (**27b**, *c* = 1.0 in trichloromethane); –75.4° (**27c**, *c* = 1.0 in trichloromethane). — FT-IR (KBr): ν = 1727 cm^{-1}. — ^{1}H-NMR ($CDCl_3$): **27a**: δ = 0.72 (d, *J* = 6.9 Hz, 6H, 10-H), 0.89, 0.91 (2d, *J* = 4.8 Hz, each 6H, 8-H, 9-H), 1.02 (s, 18H, 2x $C(CH_3)_3$), 0.80–1.10 (m, 4H), 1.20–1.70 (m, 10H), 2.18, 1.98 (2m_c, each 2H), 2.42 (s, 2H, 2´-H, 3´-H), 4.66 (ddd, *J* = 10.9 Hz, *J* = 10.8 Hz, *J* = 4.3 Hz, 2H, 1-H); **27b**: δ = 0.68, 0.70 (2d, *J* = 6.9 Hz, each 3H, 2x 10-H), 0.88, 0.90, 0.92, 0.93 (4d, *J* = 6.7 Hz, each 3H, 2x 8-H, 2x 9-H), 0.99, 1.02 (2s, each 9H, 2x $C(CH_3)_3$), 0.80–1.10 (m, 4H), 1.30–1.80 (m, 10H), 2.05–2.35 (m, 4H), 2.65, 2.70 (2d, *J* = 10.7 Hz, each 1H, 2´-H, 3´-H), 4.49, 4.51 (2ddd, *J* = 10.9 Hz, *J* = 10.8 Hz, *J* = 4.3 Hz, 2H, 2x 1-H); **27c**: δ = 0.78 (d, *J* = 6.9 Hz, 6H, 2x 10-H), 0.87, 0.89 (2d, *J* = 6.7 Hz, each 6H, 8-H, 9-H), 1.02 (s, 18H, 2x $C(CH_3)_3$), 0.80–1.10 (m, 4H), 1.20–1.70 (m, 10H), 2.05–2.25 (m, 4H), 2.41 (s, 2H, 2´-H, 3´-H), 4.64 (ddd, *J* = 10.9 Hz, *J* = 10.8 Hz, *J* = 4.3 Hz, 2H, 1-H). — ^{13}C-NMR ($CDCl_3$): **27a**: δ = 15.70 (q, C-10), 20.96, 22.14 (2q, C-8, C-9), 22.88 (t, C-3), 25.68 (d, C-7), 28.04 (q, $C(CH_3)_3$), 31.41 (d, C-5), 34.51 (t, C-4), 34.64 (s, $C(CH_3)_3$), 40.27 (t, C-6), 47.08 (d, C-2), 53.98 (d, C-2´, C-3´), 73.70 (d, C-1), 172.29 (s, C=O); **27b**: δ = 15.47, 15.64 (2q, C-10), 21.16, 21.23, 22.07 (3q, C-8, C-9), 22.68 (t, C-3), 24.90, 25.17 (2d, C-7), 28.88, 28.98 (2q, $C(CH_3)_3$), 31.41 (d, C-5), 34.07, 34.20 (2t, C-4), 34.64 (s, $C(CH_3)_3$), 40.37 (t, C-6), 47.11 (d, C-2), 53.65, 54.46 (2d, C-2´, C-3´), 75.08, 75.48 (2d, C-1), 174.25, 174.42 (2s, C=O); **27c**: δ = 15.59 (q, C-10), 21.06, 22.14 (2q, C-8, C-9), 22.71 (t, C-3), 25.07 (d, C-7), 27.97 (q, $C(CH_3)_3$), 31.44 (d, C-5), 34.62 (t, C-4), 35.10 (s, $C(CH_3)_3$), 40.86 (t, C-6), 47.09 (d, C-2), 53.73 (d, C-2´, C-3´), 74.40 (d, C-1), 172.29 (s,

C=O). — MS (GC/MS), *m/z* (%): 506 (0.4), 491 (0.4), 450 (1), 393 (20), 369 (25), 231 (95), 213 (100), 139 (36), 117 (26), 83 (43), 57 (27). — $C_{32}H_{58}O_4$ (506.8): calcd. C 75.84, H 11.53; found C 75.83, H 11.73.

22. *Di[(1R,2S,5R)-2-(tert.-butyl)-5-methylcyclohexyl] (2´R,3´R)-2´,3´-di(tert.-butyl)succinate* (**28a**) and *di[(1R,2S,5R)-2-(tert.-butyl)-5-methylcyclohexyl] (2´R,3´S)-2´,3´-di(tert.-butyl)succinate* (**28b**): 511.3 mg (1.64 mmol) of **15a/b** were electrolyzed according to the general procedure for the Kolbe electrolysis to afford 152.1 mg (0.284 mmol, 35%) **28a** and 66.2 mg (0.124 mmol, 15%) **28b** after flash chromatography and HPLC (methylene chloride: petroleum ether, 1:3). The diastereomeric ratio was determined by GLC to be **28a** : **28b** : **28c** = 5.14 : 2.00 : 0.21. The very small amount of **28c** could not be isolated.

m.p. 95-99 °C (**28a**). — $[\alpha]_D^{20}$ = –90.8° (**28a**, *c* = 1.0 in trichloromethane); –98.0° (**28b**, *c* = 0.4 in trichloromethane). — FT-IR (KBr): ν = 1726 cm^{-1}. — ^{1}H-NMR ($CDCl_3$): **28a**: δ = 0.92 (d, *J* = 6.7 Hz, 6H, 11-H), 0.94, 1.06 (2s, each 18H, $C(CH_3)_3$), 0.80-1.60 (m, 10H), 1.65, 1.84, 2.32 (3m_c, each 2H), 2.40 (s, 2H, 2´-H, 3´-H), 4.56 (ddd, *J* = 10.8 Hz, *J* = 10.6 Hz, *J* = 3.8 Hz, 2H, 1-H); **28b**: δ = 0.88 (d, *J* = 6.7 Hz, 6H, 11-H), 0.96, 0.97, 1.04, 1.08 (4s, each 9H, $C(CH_3)_3$), 0.80-1.60 (m, 10H), 1.66, 1.87, 2.33 (3m_c, each 2H), 2.57, 2.76 (2d, *J* = 6.9 Hz, each 1H, 2´-H, 3´-H), 4.55, 4.59 (2ddd, *J* = 10.8 Hz, *J* = 10.5 Hz, *J* = 3.8 Hz, each 1H, 1-H). — ^{13}C-NMR ($CDCl_3$): **28a**: δ = 22.17 (q, C-11), 26.76 (t, C-3), 28.61, 29.15 (2q, $C(CH_3)_3$), 31.44 (d, C-5), 32.59 (s, $C(CH_3)_3$), 35.08 (t, C-4), 35.15 (s, C-7), 40.57 (t, C-6), 50.11 (d, C-2), 54.05 (d, C-2´, C-3´), 76.60 (d, C-1), 172.90 (s, C=O); **28b**: δ = 22.01 (q, C-11), 26.59 (t, C-3), 29.12, 29.18, 29.35, 29.52 (4q, $C(CH_3)_3$), 31.27 (d, C-5), 32.69, 32.79 (2s, $C(CH_3)_3$), 34.74 (t, C-4), 34.61, 34.95 (2s, C-7), 40.74 (t, C-6), 50.18, 50.55 (2d, C-2), 54.69, 54.76 (2d, C-2´, C-3´), 77.10, 78.21 (2d, C-1), 173.68, 174.29 (2s, C=O). — MS (GC/MS), *m/z* (%):519 (1), 478 (1), 421 (6), 383 (2), 367 (2), 269 (14), 231 (100), 213 (82), 174 (18), 153 (46), 117

(23), 97 (66), 83 (30), 57 (93). — $C_{34}H_{62}O_4$ (534.9): calcd. C 76.35, H 11.68; found C 76.24, H 11.88.

23. *Di[(1R,2S,5R)-5-methyl-2-(1-methyl-1-phenylethyl)cyclohexyl] (2´R,3´R)-2´,3´-di(tert-butyl)succinate* (**29a**) and *di[(1R,2S,5R)-5-methyl-2-(1-methyl-1-phenylethyl)cyclohexyl] (2´R,3´S)-2´,3´-di(tert-butyl)succinate* (**29b**): 613.9 mg (1.64 mmol) of **16a/b** were electrolyzed according to the general procedure for the Kolbe electrolysis to afford 91.3 mg (0.138 mmol, 17%) **29a** and 22.8 mg (0.035 mmol, 4%) **29b** after flash chromatography and HPLC (petroleum ether(diethyl ether, 20:1). The diastereomeric ratio was determined by GLC to be **29a** : **29b** : **29c** = 7.03 : 2.00 : n.d.. The very small amount of **29c** could not be isolated nor determined.

m.p. 144–148 °C (**29a**); 84–88 °C (**29b**). — $[\alpha]_D^{20}$ = –50.0° (**29a**, *c* = 1.0 in trichloromethane); –33.8° (**29b**, *c* = 1.0 in trichloromethane). — FT-IR (KBr): ν = 1720 cm^{-1}. — ^{1}H-NMR ($CDCl_3$): **29a**: δ = 0.89 (d, *J* = 6.5 Hz, 6H, 10-H), 0.84 (s, 18H, $C(CH_3)_3$), 1.15, 1.33 (2s, each 6H, 8-H, 9-H), 0.80–1.70 (m, 12H), 1.75 (s, 2H, 2´-H, 3´-H), 2.08, 2.29 ($2m_c$, each 2H), 4.48 (ddd, *J* = 10.5 Hz, *J* = 10.4 Hz, *J* = 3.6 Hz, 2H, 1-H), 7.10–7.35 (m, 10H, C_6H_5); **29b**: δ = 0.86 (d, *J* = 6.4 Hz, 6H, 10-H), 0.99, 1.08 (2s, each 9H, $C(C\mathit{H_3})_3$), 1.24, 1.27, 1.47, 1.52 (4s, each 3H, 8-H, 9-H), 0.70–1.60 (m, 12H), 2.40, 2.53 (2d, *J* = 6.9 Hz, 2H, 2´-H, 3´-H), 1.90–2.60 (m, 4H), 4.62, 4.63 (2ddd, *J* = 10.5 Hz, *J* = 10.4 Hz, *J* = 3.6 Hz, each 1H, 1-H), 7.10–7.35 (m, 10H, C_6H_5). — ^{13}C-NMR ($CDCl_3$): **29a**: δ = 22.14 (q, C-10), 25.71, 27.26 (2q, C-8, C-9), 27.13 (t, C-3), 28.27 (q, $C(\mathit{C}H_3)_3$), 31.54 (d, C-5), 34.81 (s, $\mathit{C}(CH_3)_3$), 35.01 (t, C-4), 39.66 (s, C-7), 40.40 (t, C-6), 50.11 (d, C-2), 52.80 (d, C-2´, C-3´), 76.43 (d, C-1), 124.99, 125.66, 127.88 (3d, =C-H_{phenyl}), 152.18 (s, =C–C_{phenyl}), 173.17 (s, C=O); **29b**: δ = 21.87 (q, C-10), 22.37, 23.98, 28.85, 30.51 (4q, C-8, C-9), 27.40, 27.58 (2t, C-3), 29.01, 29.40 (2q, $C(\mathit{C}H_3)_3$), 31.41 (d, C-5), 34.47, 34.92 (2s, $\mathit{C}(CH_3)_3$), 34.59 (t, C-4), 40.18, 40.50 (2s, C-

7), 40.80 (t, C-6), 50.51, 51.01 (2d, C-2), 53.99, 54.43 (2d, C-2´, C-3´), 77.17, 78.17 (2d, C-1), 125.14, 125.20, 125.52, 125.58, 127.96, 127.99 (6d, =*C*-H_{phenyl}), 151.38, 151.49 (2s, =*C*–C_{phenyl}), 173.54, 174.38 (2s, C=O). — MS (DEI), *m/z* (%): 540 (12), 445 (3), 422 (5), 325 (17), 231 (25), 214 (19), 127 (16), 119 (60), 118 (30), 85 (58), 71 (79), 57 (100). — HR MS (NH_3-DCI): $C_{44}H_{66}O_4$: calcd. 659.5039; found 659.5057{M +H^+}.

24. (1*R*,2*S*,5*R*)-5-methyl-2-(2-phenylpropan-2-yl) cyclohexyl (2-*R*)-4,4-dimethyl-2-phenylpentanoate (*R*-**30c**) [SI-2]

Ester **30c** was prepared by esterification of 2-phenyl-4,4-dimethylpentanoic acid (**32**) with the auxiliary **3**. Acid **32** was prepared in 94% yield by addition of *tert*-butyl lithium to styrene and carboxylation of the intermediate benzyl lithium compound with carbon dioxide [SI-2].

In a two-necked flask with reflux condenser annealed to 80 °C under argon acid **32** (464 mg, 2.25 mmol) and thionyl chloride (0.3 mL) dissolved in absolute dichloromethane were refluxed for 3 h. After stirring for further 2 h at rt excess thionylchloride was distilled off under reduced pressure and the colourless residue was dissolved at −10 °C in dichloromethane (10 mL). Thereafter a solution of 4-dimethylaminopyridine (25 mg, 0.2 mmol, triethylamine (0.3 g, 3.00 mmol) and 8-phenylmenthol (**3**, 472 mg, 2.04 mmol) in abs. dichloromethane (10 mL) were added slowly and then the solution was stirred for 3 h at −10 °C. After stirring for further 16 h at rt, a sat. sodium hydrogen carbonate solution (10 mL) was slowly added dropwise and then the organic phase was separated. The aqueous phase was extracted with dichloromethane (2 × 10 mL) and the combined organic phases were dried ($MgSO_4$). Finally the solvent was rotaevaporated and the crude product purified by flash chromatography (PE/E 15:1). From the combined fractions, which contained both diastereomers the solvent was rotaevaporated to yield 410 mg and the residue was dissolved in ethanol (1 mL). After 24 h the *R*-isomer precipitated in white needles. The filtered crystals

were recrystallized twice from ethanol. The (*S*)-isomer could be isolated by way of middle pressure chromatography (PE/E 30:1).

Yield: 95 mg (0.23 mmol, 11%) *R*-**30c**; for the configuration of *R*-**30c** see [SI-2]; 149 mg (0.35 mmol, 17%) (*S*)-**30c**;

R_f-value: 0.49 (*R*-**30c**), 0.51 ((*S*)-**30c)** (PE : E = 30 :1).

(*R*-**30c**):

$[\alpha]^{20}_D$: -41.7° (c=0.9, $CHCl_3$)

FT-IR (film): ν = 3084, 3009, 2949, 2921, 2854 (s), 1719 (s),1599, 1492 (m), 1387, 1366 (m), 1206, 1152 (s)765, 730, 701(s).

^{1}H NMR ($CDCl_3$): δ(ppm) = 0.84 (d, ^3J = 6.4 Hz, 3 H), 0.86 (s,9 H), 0.97, 1.01 (2s, each 3H), 0.65-0.94, 1.18-1.51 (2m, 6H), 1.55 (dd, ^3J =4.5 Hz, ^2J =14.1 Hz, 1H), 1.79-1.88 (m,1H), 1.93-2.03 (m, 1H), 2.22 (dd, ^3J = 8.1 Hz, ^2J =14.1 Hz, 1H), 3.41 (dd,^3J = 4.5 Hz, ^3J = 8.1 Hz, 1H) 4.66 (ddd, ^3J = 4.5 Hz, ^3J =8.1 Hz, 1H), 4.66 (ddd, ^3J = 4.5 Hz, ^3J= 10.5 Hz, ^3J=10.5 Hz,1H),7.04-7.21 (m, 10H).

^{13}C NMR ($CDCl_3$): δ (ppm) = 21.8 (q), 24.3, 29.3 (2q), 27.3 (t), 29.6 (q), 31.1 (s), 31.3 (), 34.6 (t), 40.1 (s), 41.4 (t), 48.1 (t), 48.7 (d), 50.6 (d), 75.7 (d), 125.2, 125.7, 126.9, 127.9, 128.3, 128.4 (6d), 140.6 (s), 1740 (s).

MS (GC/MS-coupling) *m/z* (%) = 420 (1), 301 (10), 214 (38), 119 (100), 105 (24), 91 (19) 57 (19).

$C_{24}H_{40}O_2$ (420.64) calc. C 82.81, H 9.58; found C 82.89, H 9.68.

Cleavage of the auxiliary from (*R*-**30c)** **[**SI-2**]**

(2-*R*)-4,4_Dimethyl-2-phenylpentanol (*R*-**31c**): In an annealed two neck flask under argon to a suspension of lithium aluminium hydride (11.3 mg, 0.310 mmol) in THF (2 mL) was added dropwise at 0 °C to a solution of ester ***R*-30c** (82.3 mg, 0.196 mmol) in 3 mL THF and the mixture was then stirred for 60 h at rt. Then a sat. ammonium chloride solution (0.5 mL) and a sat. sodium chloride solution (5 mL) were added. After separating the org. phase, the aqueous phase was extracted with diethyl ether (4 × 5 mL) and then the combined org. phases were dried ($MgSO_4$). After rotaevaporating the solvent the crude oily product was purified by flash chromatography (PE/E 4:1) to obtain alcohol *R*-**31c** (35.1 mg, 0.182 mmol, 93%) and 8-phenyl menthol (**3**) (37.7 mg, 0.163 mmol, 83%).

(2*R*)-4,4-Dimethyl-2-phenylpentanol (*R*-**31c**)

R_f-value: 0.13 (PE: E = 4 :1); mp. 49-50 °C; $[\alpha]^{20}_D$: -4.2° (c=1.02, $CHCl_3$).

FT-IR (KBr): ν (cm^{-1}) = 3246 (br), 3085, 3027 (m), 2956, 2935, 2868 (s), 618, 494 (w), 1384, 1365 (m), 760, 702 (s).

^{1}H NMR ($CDCl_3$): δ (ppm) = 0.82 (s, 9H), 1.52 (d,^3J= 3.6 Hz, ^2J = 14.1 Hz, 1H), 1.66 (dd, ^3J = 8.3 Hz, ^2J= 14.1 Hz, 1H), 2.87 (m, 1H), 3.58 (dd, ^3J= 8.3 Hz, ^2J = 10.7 Hz, 1H), 3.66 (dd, ^3J = 6.0 Hz, ^2J = 10.7 Hz, 1H), 7.18-7.35 (m).

^{13}C NMR ($CDCl_3$) δ (ppm) = 28.0 (q), 31.1 (s), 45.4 (d), 45.8 (t), 69.0 (t), 126.5, 128.25, 128.6 (3d), 144.2 (s).

MS (GC/MS-coupling): *m/z* (%) = 192 (18), 162 (26), 121 (7), 105 (42), 91 (23), 57 (100).

$C_{13}H_{20}O$ (192.30) calc. C 81.19, H 10.49; found: C81.24, H 10.51.

25. *X-ray crystal structure analysis of* **23b**: formula $C_{22}H_{42}O_2$, M = 338.56, 0.50 × 0.25 × 0.20 mm, a = 9.634(1), b = 10.240(2), c = 11.754(2) Å, β = 102.74(1)°, V = 1131.0(3) Å^3, ρ_{calc} =

0.994 g · cm^{-3}, μ = 4.62 cm^{-1}, empirical absorption correction via φ scan data (0.895 ≤ C ≤ 0.998), Z = 2, monoclinic, space group P2_1 (No. 4), λ = 1.54178 Å, T = 223 K, $\omega/2\theta$ scans, 2580 reflections collected (+h, +k, ±l), [(sinθ)/λ] = 0.62 Å^{-1}, 2436 independent and 1738 observed reflections [$I \geq 2\ \sigma(I)$], 227 refined parameters, R = 0.050, wR^2 = 0.122, max. residual electron density 0.17 (-0.18) e Å^{-3}, Flack parameter 0.0(4), hydrogens calculated and refined as riding atoms.

X-ray crystal structure analysis of **28a**: formula $C_{34}H_{62}O_4$, M = 534.84, 0.50 × 0.25 × 0.15 mm, a = 11.751(1), b = 11.222(1), c = 27.374(4) Å, β = 95.93(1)°, V = 3590.5(7) Å^3, ρ_{calc} = 0.989 g · cm^{-3}, μ = 4.81 cm^{-1}, empirical absorption correction via φ scan data (0.971 ≤ C ≤ 0.998), Z = 4, monoclinic, space group P2_1 (No. 4), λ = 1.54178 Å, T = 223 K, $\omega/2\theta$ scans, 7861 reflections collected (±h, -k, -l), [(sinθ)/λ] = 0.62 Å^{-1}, 7701 independent and 6165 observed reflections [$I \geq 2\ \sigma(I)$], 714 refined parameters, R = 0.043, wR^2 = 0.115, max. residual electron density 0.18 (-0.17) e · Å^{-3}, Flack parameter 0.1(2), hydrogens calculated and refined as riding atoms.

X-ray crystal structure analysis of **29a**: formula $C_{44}H_{66}O_4$, M = 658.97, 0.40 × 0.30 × 0.20 mm, a = 10.860(1), b = 16.786(1), c = 22.380(2) Å, V = 4079.8(6) Å^3, ρ_{calc} = 1.073 g · cm^{-3}, μ = 5.11 cm^{-1}, empirical absorption correction via φ scan data (0.976 ≤ C ≤ 0.999), Z = 4, orthorhombic, space group P$2_12_12_1$ (No. 19), λ = 1.54178 Å, T = 223 K, $\omega/2\theta$ scans, 4626 reflections collected (-h, -k, -l), [(sinθ)/λ] = 0.62 Å^{-1}, 4626 independent and 4206 observed reflections [$I \geq 2\ \sigma(I)$], 446 refined parameters, R = 0.037, wR^2 = 0.104, max. residual electron density 0.15 (-0.12) e · Å^{-3}, Flack parameter 0.1(2), hydrogens calculated and refined as riding atoms.

References

[SI-1] Ihara, M.; Takahashi, M.; Taniguchi, N.; Yagui, K.; Fukumoto, K. *J. Chem. Soc. Perkin. Trans. I* **1989**, 897-903.

[SI-2] Nguyen, P. Q., Master Thesis, University of Münster **1998**, p.58.
